# Physicochemical Approach to Understanding the Structure, Conformation, and Activity of Mannan Polysaccharides

**DOI:** 10.1021/acs.biomac.0c01659

**Published:** 2021-03-17

**Authors:** Angela Casillo, Antonio Fabozzi, Irene Russo Krauss, Ermenegilda Parrilli, Caroline I. Biggs, Matthew I. Gibson, Rosa Lanzetta, Marie-Sousai Appavou, Aurel Radulescu, Maria L. Tutino, Luigi Paduano, Maria M. Corsaro

**Affiliations:** † Department of Chemical Sciences, University of Naples “Federico II”, Complesso Universitario Monte S. Angelo, Via Cintia 4, 80126 Naples, Italy; ‡ CSGI - Consorzio per lo Sviluppo dei Sistemi a Grande Interfase, Florence 50019, Italy; § Department of Chemistry, University of Warwick, Coventry CV4 7AL, U.K.; ∥ Warwick Medical School, University of Warwick, Coventry CV4 7AL, U.K.; ⊥ Jülich Centre for Neutron Science, Garching Forschungszentrum, Lichtenbergstrasse 1, D-857478 Garching bei München, Germany

## Abstract

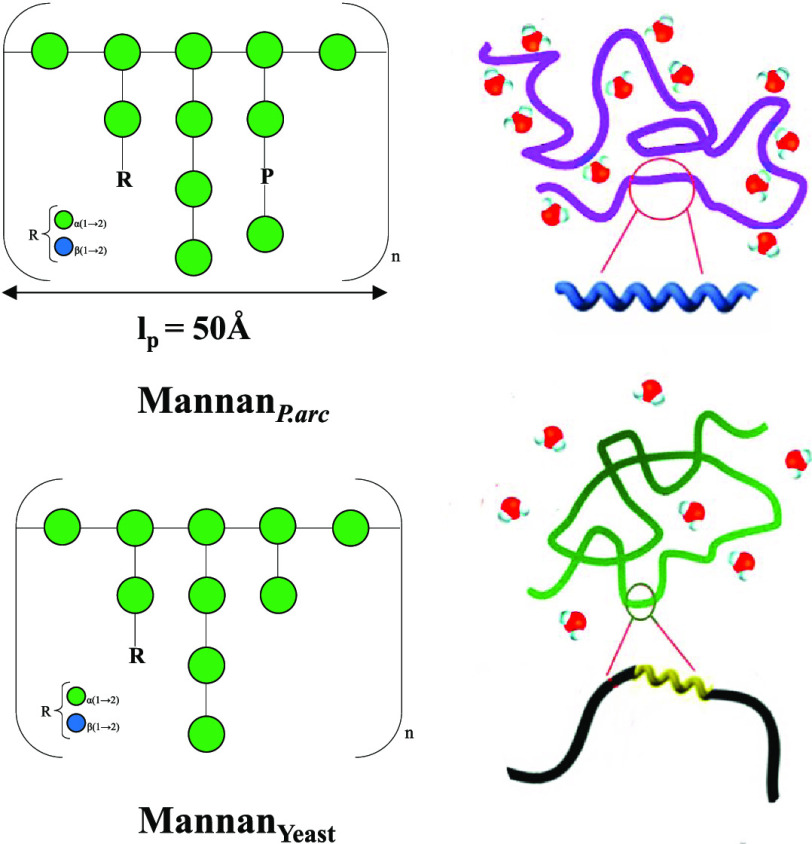

Extracellular polysaccharides are widely produced by bacteria, yeasts, and algae. These polymers are involved in several biological functions, such as bacteria adhesion to surface and biofilm formation, ion sequestering, protection from desiccation, and cryoprotection. The chemical characterization of these polymers is the starting point for obtaining relationships between their structures and their various functions. While this fundamental correlation is well reported and studied for the proteins, for the polysaccharides, this relationship is less intuitive. In this paper, we elucidate the chemical structure and conformational studies of a mannan exopolysaccharide from the permafrost isolated bacterium *Psychrobacter arcticus* strain 273-4. The mannan from the cold-adapted bacterium was compared with its dephosphorylated derivative and the commercial product from *Saccharomyces cerevisiae*. Starting from the chemical structure, we explored a new approach to deepen the study of the structure/activity relationship. A pool of physicochemical techniques, ranging from small-angle neutron scattering (SANS) and dynamic and static light scattering (DLS and SLS, respectively) to circular dichroism (CD) and cryo-transmission electron microscopy (cryo-TEM), have been used. Finally, the ice recrystallization inhibition activity of the polysaccharides was explored. The experimental evidence suggests that the mannan exopolysaccharide from *P. arcticus* bacterium has an efficient interaction with the water molecules, and it is structurally characterized by rigid-rod regions assuming a 14-helix-type conformation.

## 1 Introduction

Microbial extracellular polysaccharides (EPSs) are high-molecular-weight polymers^1^ surrounding the cells or secreted in the growth medium^2^ and are produced by bacteria, algae, and yeasts. These polymers are the most abundant components of biofilm, assuming a crucial role in the pathogenic bacteria.^3^ The EPSs participate in the adhesion of bacteria to many surfaces, play a role in the protection of the microorganisms, and boost the biochemical interactions between the bacteria and the surrounding environment.^2,4,5^

Although the variety of exopolysaccharides that can be found in nature is substantial, the biotechnological application of these polymers is often limited due to the lack of information regarding their structure and biological activity. To our knowledge, only a few papers have described how the structural features of the exopolysaccharides are responsible for such activities.^6,7^ Therefore, besides the investigation of the primary structure of the polysaccharide, the study of the physical/chemical properties and the shape the polysaccharides adopt in solution could represent the key to understand their bioactivity.

The mannose-type polysaccharides, usually referred to as mannans, are widely distributed in nature as part of hemicelluloses in plant tissue^8,9^ as well as constituents of glycoproteins in yeast cell walls.^10^ Mannans differ significantly in their structure: the main backbone can consist of α-1,6-linked d-mannose residues or β-1,4-linked d-mannose, and could be linear or branched polysaccharides. The degree of branching and the type of substituents represent another source of variability. Mannan polysaccharides have been found to be mainly delivered in the growth medium of pathogenic fungi^11^ and in some cold-adapted bacteria.^12−14^ In the case of the polymers produced by fungi, such as, for example, *Candida albicans*, it has been demonstrated that they are constituents of biofilm and might have a role in the biofilm adhesion and in the drug resistance mechanisms.^15^ The role assumed by the mannans for the cold-adapted bacteria has not been well defined yet, since only in one case, they have been suggested to be cryoprotectants.^13^

Few physicochemical methods applied to the conformational study of polysaccharides are available, such as static light scattering^16^ and atomic force microscopy.^17^ Some of them, such as UV circular dichroism (CD), are adopted from protein analyses, but this technique is much less developed for analysis of polysaccharides than for proteins and nucleic acids.^18^ The CD technique is often hampered by the absence of suitable chromophores in most natural polysaccharides.^19^ To the best of our knowledge, all of these techniques have never been applied to study mannan polysaccharide.

Here, we report the complete structural characterization and a physicochemical conformational study of a mannan extracellular polysaccharide produced by the cold-adapted permafrost isolate *Psychrobacter arcticus* 273-4.^20^ The investigation of mannan polysaccharides is often restricted to a shallow analysis, and the absence of a well-defined repeating unit hampers the conformational study through a molecular dynamic calculation approach. In the present work, a physicochemical approach has been undertaken by exploitation of small-angle neutron scattering (SANS), dynamic and static light scattering (DLS and SLS, respectively), CD, and cryo-transmission electron microscopy (cryo-TEM), a combination of techniques that is not usually employed for analysis of polysaccharides. We compared all of the results with the commercial mannan from *Saccharomyces cerevisiae* and with the dephosphorylated *P. arcticus* mannan. Finally, following on our previous papers reporting the weak ice recrystallization inhibition (IRI) activity of extracellular polysaccharides from cold-adapted bacteria,^6,7^ the IRI response of these mannan polysaccharides obtained from different sources was evaluated.

## 2 Experimental Details

### 2.1 Extraction and Purification

*P. arcticus* strain 273-4 was isolated from permafrost soil located in Siberia. Shake flask cultivation was performed at 4 °C, in Luria-Bertani broth added of 5% of NaCl, and under aerobic condition. When the liquid cultures reached late exponential phase (about 72 h, OD_600_ = 4), cells were collected by centrifugation for 15 min at 7000 rpm at 4 °C. The exopolysaccharide content was separated from the supernatant through the addition of three volumes of cold ethanol followed by 72 h of precipitation at −20 °C. The precipitated was then collected after centrifugation at 8000 rpm at 4 °C, dissolved in water, and lyophilized (0.5 g L^–1^). The mixture was purified through a Sephacryl S400 gel filtration column (flow rate, 15 mL h^–1^, 0.75 cm × 90 cm) (S400 HR, Sigma-Aldrich, Italy) eluted with 50 mM ammonium hydrogen carbonate. The chromatographic system was equipped with a Knauer RI detector 2300 and a Gilson FC203B fraction collector. The obtained high-molecular-weight fraction was freeze-dried (3.3%). The protein concentration was estimated using the Bradford method (Bio-Rad).

### 2.2 Chemical Analysis

The sugar composition was determined by gas chromatography-mass spectrometry (GC-MS) analysis after derivatization of the sample as acetylated methyl glycosides (AMG).^21^ Briefly, the sample (1 mg) was dissolved in 1 mL of 1.25 M MeOH/HCl solution (Sigma-Aldrich, Italy) and kept at 80 °C for 16 h. After the methanolysis reaction, the sample was evaporated to dryness and dissolved in 200 μL of pyridine and 100 μL of acetic anhydride (100 °C for 30 min). The obtained sample was evaporated, dissolved in chloroform, and extracted three times with water. The final organic phase was evaporated, dissolved in acetone, and analyzed by GC-MS. The linkage position was obtained after derivatization of the sample in partially methylated acetylated alditols.^22^ The sample was methylated with 100 μL of CH_3_I and then hydrolyzed at 120 °C with trifluoroacetyl (TFA) 2 M for 2 h. After neutralization, it was reduced with NaBD_4_ and finally acetylated and injected into the GC-MS. All of the derivative samples were analyzed using an Agilent Technologies gas chromatograph 7820A equipped with a mass selective detector 5977B and an HP-5 capillary column (Agilent, 30 m × 0.25 mm i.d.; flow rate, 1 mL min^–1^, He as carrier gas). Acetylated methyl glycosides and partially methylated alditol acetates were analyzed using the following temperature program: 140 °C for 3 min, 140 → 240 °C at 3 °C min^–1^ and 90 °C for 1 min, 90 → 140 °C at 25 °C min^–1^, 140 → 200 °C at 5 °C min^–1^, 200 → 280 °C at 10 °C min^–1^, at 280 °C for 10 min.

### 2.3 HF Hydrolysis

Purified EPS (40 mg) from *P. arcticus* (Mannan_P.arc_) was hydrolyzed with 4 mL of 48% HF at 4 °C for 48 h.^23^ The mixture was neutralized and purified on a Sephacryl S400 gel filtration column (flow rate, 13.8 mL h^–1^, 0.75 cm × 90 cm) (S400 HR, Sigma-Aldrich, Italy) eluted with 50 mM ammonium hydrogen carbonate. The obtained fraction was analyzed by ^1^H NMR spectroscopy, to confirm that the reaction occurred.

### 2.4 NMR Spectroscopy

One-dimensional (1D) and two-dimensional (2D) NMR experiments were acquired in D_2_O at a 600 MHz Bruker (Bruker Italia, Italy) instrument equipped with a cryogenic probe. The spectra were recorded at 298 K using acetone as external standard (δ_H_ = 2.225 ppm; δ_C_ = 31.45 ppm). Spectra were processed and analyzed using Bruker Top Spin 3.1 software. Double-quantum-filtered phase-sensitive correlation spectroscopy (^1^H–^1^H DQF-COSY), total correlation spectroscopy (^1^H–^1^H TOCSY), and nuclear Overhauser enhancement spectroscopy (^1^H–^1^H NOESY) experiments were executed using 256 FIDs of a 2028 complex point. TOCSY and NOESY experiments were recorded with a mixing time of 100 ms. Heteronuclear single quantum coherence (^1^H–^13^C DEPT-HSQC) and heteronuclear multiple bond correlation (^1^H–^13^C HMBC) experiments were acquired with 512 FIDs of a 2048 complex point. ^1^P- and ^1^H–^31^P HMBC spectra were recorded at 298 K using a Bruker Ascend 400 MHz spectrometer. The ^1^H–^31^P HMBC experiment was acquired with 512 FIDs of a 2048 complex point.

### 2.5 Static and Dynamic Light Scattering (SLS and DLS) Characterization

Static and dynamic light scattering (SLS and DLS, respectively) measurements were performed at scattering angle θ = 90°, using a homemade instrument composed of a photocor compact goniometer, an SMD 6000 Laser Quantum 50 mW light source operating at 532.5 nm, a photomultiplier (PMT120-OP/B), and a correlator (Flex02-01D) from Correlator.com. Measurements were performed at 4 and 25 °C with the temperature controlled by means of a thermostat bath.^24^ DLS measurements were performed on both diluted (0.2, 0.2, and 0.1 mg mL^–1^, for Mannan_P.arc_, Mannan_yeast_, and Mannan_P.arc_HF_, respectively) and concentrated (1.0 and 6.0 mg mL^–1^) polysaccharide samples. For SLS measurements, stock solutions of pure Mannan_yeast_, Mannan_P.arc_HF_, and Mannan_P.arc_, at 2.0, 2.0, and 1.0 mg mL^–1^, respectively, were used. Deionized water filtered through a 0.22 μm membrane was used in all of the cases. The mass-averaged molecular weight *M*_w_ and the second virial coefficient *B* of each polysaccharide were determined by means of Zimm plot analysis
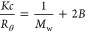
1where *c* is the sample mass concentration;  with *n*_0_ = 1.33, the refractive index of water, d*n*/d*c* = 0.185, the refractive index increment with concentration;^25,26^*N*_A_ is Avogadroʼs number, λ is the laser wavelength in vacuum, and *R*_θ_ is the excess Rayleigh ratio at 90°. The value of *R*_θ_ was obtained from , where *I*_s_ is the scattered intensity of the solution, *I*_s,0_ is the scattered intensity of water, *I*_s,R_ is the scattering intensity of toluene (the standard), and *n*_R_ = 1.496 and *R*_θ,R_ = 2.85 10^–5^ cm^–1^ are the refractive index and the Rayleigh ratio of toluene, respectively.^27^

In the case of DLS, the data were treated with CONTIN: namely, the measurements, at least five independent measurements for each sample, were analyzed with “Precision Deconvolve”, a program based on the approach of Benedek and Lomakin.^28^ The proper diffusion coefficients were determined through a final assessment by the “regularization” procedure.^29^ Diffusion coefficients were then employed to calculate hydrodynamic radii by means of the Stokes–Einstein relation
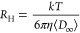
2where *k* is the Boltzmann constant, *T* is the absolute temperature, and η is the medium viscosity, whose mean value was assumed to be 0.89 cP for each aqueous mixture.

### 2.6 Surface Tension Titration

The surface tension, γ, of aqueous mixtures of Mannan_P.arc_, Mannan_Yeast_, and Mannan_P.arc_HF_ was measured at 25 °C with a Sigma 70 tensiometer (KSV, Stockholm, Sweden) using the Du Noüy ring method as described elsewhere.^30^ γ was correlated with the force required to raise the ring from the surface of the air/liquid interface. Successive aliquots of a stock polysaccharide solution were added to the vessel with a known volume of water. After each aliquot addition, the sample was mixed using a magnetic stirrer and allowed to equilibrate 3 min prior to measuring the surface tension.

### 2.7 Small-Angle Neutron Scattering (SANS)

SANS measurements of the samples of Mannan_P.arc_ and Mannan_Yeast_ were performed with the KWS2 instrument located at the Heinz Meier Leibtnitz Source, Garching Forschungszentrum (Germany).^31^ Neutrons with a wavelength spread Δλ/λ ≤ 0.2 were used. A two-dimensional array detector at different wavelength, collimation, sample-to-detector distance combinations measured neutrons scattered from the samples. We chose configurations that allowed collecting data in a *q* range of 0.0018–0.45 Å^–1^. The samples were contained in a closed-quartz cell, to prevent the solvent evaporation, and all measurements were performed at 25 °C. D_2_O samples at 1.0 and 2.0 mg mL^–1^ concentrations for Mannan_P.arc_ and Mannan_Yeast_, respectively, were analyzed. Each measurement lasted a period sufficient to obtain ∼2 million counts.

Raw SANS data were corrected for background and empty cell scattering. Detector efficiency correction, radial average, and transformation to absolute scattering cross sections d∑/dΩ were made with a secondary plexiglass standard.^32,33^ The absolute scattering cross-sectional data d∑/dΩ were plotted as a function of *q*.

### 2.8 Cryogenic Transmission Electron Microscopy (cryo-TEM)

Cryogenic transmission electron microscopy (cryo-TEM) images were carried out at the Heinz Maier-Leibnitz Zentrum, Garching, Germany, on a JEOL 200 kV JEM-FS2200 with a field emission gun (FEG). Samples for TEM were prepared by placing a 5 μL drop of a 6.3 mg mL^–1^ solution of Mannan_P.arc_ or a 6 mg mL^–1^ solution of Mannan_Yeast_ on a Quantifoil Multi A carbon-coated copper grid. After a few seconds, excess solution was removed by blotting with filter paper. The sample was cryo-fixed by rapidly immersing into liquid ethane at −180 °C in a cryo-plunge (EMGP Leica GmbH). The specimen was inserted into a cryo-transfer holder (HTTC 910, Gatan, Munich, Germany) and transferred to a JEM 2200 FS EFTEM instrument (JEOL, Tokyo, Japan). Examinations were carried out at temperatures around −180 °C. The transmission electron microscope was operated at an acceleration voltage of 200 kV. Zero-loss-filtered images were taken under reduced-dose conditions (<10 000 e^–^ nm^–2^). All images were recorded digitally by a bottom-mounted 16 bit CMOS camera system (TemCam-F216, TVIPS, Munich, Germany). To avoid any saturation of the gray values, all of the measurements were taken with intensity below 15 000, considering that the maximum value for a 16 bit camera is 2^16^. Images have been taken with EMenu 4.0 image acquisition program (TVIPS, Munich, Germany) and processed with a free digital imaging processing system ImageJ.^34,35^

### 2.9 Circular Dichroism

Circular dichroism (CD) spectra were recorded at 4, 20, and 37 °C using a Jasco J-715 spectropolarimeter equipped with a Peltier thermostatic cell holder (Model PTC-348WI). CD measurements were carried out in the 250–190 nm range, using a 0.1 cm path length cell and polysaccharide solutions at 0.5 mg mL^–1^ concentration in water, with 0.5 nm data pitch, 2 nm bandwidth, and 20 nm min^–1^ scanning speed. Each spectrum was obtained as the average of three scans.

### 2.10 Ice Recrystallization Inhibition (IRI) Assay

A 10 μL droplet of sample in phosphate-buffered saline (PBS) solution was dropped from 1.4 m onto a glass microscope coverslip, which was placed on top of an aluminum plate cooled to −78 °C using dry ice. The droplet froze instantly upon impact with the plate, spreading out and forming a thin wafer of ice. This wafer was then placed on a liquid nitrogen-cooled cryostage held at −8 °C. The wafer was then left to anneal for 30 min at −8 °C. The number of crystals in the image was counted using ImageJ, and the area of the field of view divided by this number of crystals gives the average crystal size per wafer and is reported as a percentage (%) of area compared to PBS control.

## 3 Results

### 3.1 Mannan Purification and Chemical Analyses

*P. arcticus* 273-4 was grown at 4 °C as already reported.^22^ After centrifugation, the cells were removed and the supernatant was incubated at −20 °C for 72 h with three volumes of cold ethanol. The precipitate was then separated from the supernatant by centrifugation at 4 °C, redissolved in water and freeze-dried.

**Scheme 1:**
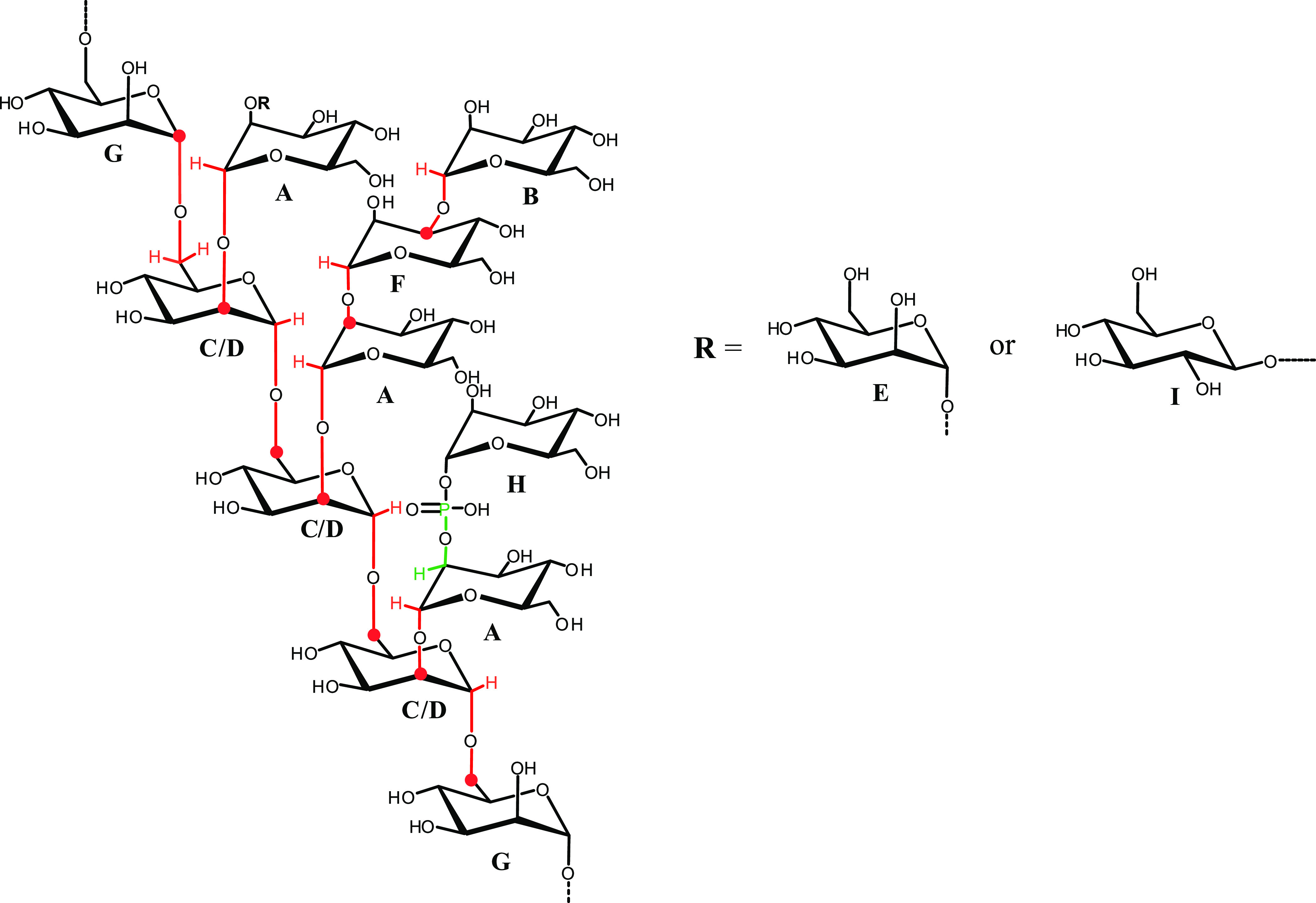
Schematic Description of Mannan_P.arc_ The ^1^H–^13^C NMR long-range correlations are highlighted in red, while those of ^1^H–^31^P are in green.

The mixture was purified through a gel filtration chromatography column, using ammonium hydrogen carbonate as an eluent. Two main peaks were obtained: first, named fraction A, containing the highest-molecular-weight compounds, and second, named fraction B, containing growth medium components (Figure S1). The GC-MS glycosyl analysis as AMG of fraction A indicated the occurrence of mannose (Man) and glucose (Glc). The analysis of partially methylated acetylated alditols (PMAA) revealed the occurrence of terminal nonreducing Man (t-Man), terminal nonreducing Glc (t-Glc), 2-substituted Man (2-Man), 3-substituted Man (3-Man), 6-substituted Man (6-Man), and 2,6-disubstituted Man units (2,6-Man) (see Scheme 1).

The AMG analysis was also performed on the commercial mannan from the yeast *S. cerevisiae* (Mannan_Yeast_), revealing the presence of mannose and glucose. Furthermore, the linkage analysis revealed the same points of attachment as in *P. arcticus* polysaccharide.^36^

Both Mannan_P.arc_ and Mannan_Yeast_ were tested for the presence of proteins by the Bradford assay. No proteins were detected in both samples.

### 3.2 NMR Study

#### 3.2.1 Mannan_P.arc_

To entirely characterize Mannan_P.arc_ polysaccharide, the complete set of 2D NMR experiments was performed (Figures S2–S7). The ^1^H–^13^C DEPT-HSQC experiment (Figure S2 and Table 1) confirmed the occurrence of different anomeric cross-peaks at δ 5.19/101.8 (**A**), 5.04/103.5 (**B**), 5.01/99.4 (**C**), 4.99/99.4 (**D**), 4.94/103.4 (**E**), 4.93/103.5 (**F**), 4.79/100.6 (**G**), 5.34/97.5 (**H**) ppm, all belonging to mannose units, and the signal at δ 4.36/102.9 ppm (**I**) attributable to the glucose residues.

**Table 1:** ^1^H and ^13^C NMR Assignments of the Mannan_P.arc_^a^

sugar residue	^1^H/^13^C (ppm) ^1^*J*_C1, H1_					
	1	2	3	4	5	6
**A**	5.19	4.01	3.80	3.62	3.66	3.64–3.80
2-Man*p*	101.8	79.8	71.5	68.1	74.4	62.3
	175					
**B**	5.04	3.96	3.75	3.53	3.66	3.64–3.78
t-Man*p*	103.5	71.3	71.6	68.2	74.5	62.4
	176					
**C**	5.01	3.93	3.83	3.73	3.72	3.57–3.91
2,6-Man*p*	99.4	80.0	71.4	67.8	72.1	66.9
	180					
**D**	4.99	3.91	3.81	3.73	3.72	3.57–3.91
2,6-Man*p*	99.4	80.0	71.5	67.8	72.1	66.9
	180					
**E**	4.94	3.96	3.70	3.53	3.66	3.64–3.78
t-Man*p*	103.4	71.3	71.7	68.1	74.4	62.4
	174					
**F**	4.93	4.11	3.85	3.64	3.70	3.64–3.78
3-Man*p*	103.5	70.9	79.2	67.6	72.5	62.4
	174					
**G**	4.79	3.88	3.72	3.59	3.63	3.67–3.84
6-Man*p*	100.6	71.3	72.1	68.1	72.1	66.7
	172					
**H**	5.34	3.91	3.88	3.68	n.d	n.d
1-*P* Man*p*	97.5	71.8	71.3	67.6		
	174					
**I**	4.36	3.17	3.45	3.69	3.67	3.64–3.78
t-Glc*p*	102.9	74.0	74.9	71.8	77.5	62.4
	163					

a Spectra were recorded in D_2_O at 298 K at 600 MHz using acetone as external standard (δ_H_/δ_C_ 2.25/31.45 ppm).

The anomeric proton and carbon chemical shifts, together with the ^1^*J*_C1,H1_ values obtained from the coupled F2-coupled DEPT-HSQC experiment allowed us to assign the configurations α and β for mannose and glucose units, respectively (Table 1 and Figure S3). The results obtained from methylation analysis and the correspondence of chemical shifts with those reported in the literature^12,36,37^ support the hypothesis of a sugar backbone consisting of α-(1→6)-linked mannopyranose units branched at C-2. The branches are constituted by 2- and/or 3-linked mannose units ending with mannose or glucose, as deduced from NMR data (Table 1). The presence of an α-(1→6) backbone was suggested by a long-range scalar connectivity between both anomeric proton signals at δ 5.01 and δ 4.99 ppm of residues **C** and **D**, respectively, with a carbon signal at δ 66.9 ppm (Table 2, Scheme 1 and Figure S4). NOE contacts between both H1-**C** and H1-**D** with H6-**C** confirmed this hypothesis (Table 2 and Figure S5). The finding of both signals of C-2 of **C** and **D** at δ 80.0 ppm indicated their substitution. Residue **A** substituted both residues **C** and **D**, as revealed by the long-range scalar correlation of H1-**A** and C2-**C**/C2-**D** (Scheme 1). Moreover, the chemical shift value of C-2 of **A** was downfield-shifted at δ 79.8 ppm, revealing its substitution. The different length of branching is suggested by the different substitution of **A**. Indeed, in some branches, the residue **A** is substituted by the terminal mannose **E**, as indicated by both NOE contacts and long-range connectivity (Table 2 and Scheme 1), whereas in others, the length of the branch is longer, as suggested by the linkage of the 3-substituted mannose **F** to residue **A**. This is confirmed by the long-range correlation between H1-**F** and C2-**A** (Table 2 and Scheme 1). The anomeric proton of **B** gave long-range scalar connectivity with C3-**F**, revealing that residue **F** is substituted by a terminal mannose **B**. Finally, glucose **I** occupies the terminal position of some branches, as suggested by the NOE contact between H1-**I** and H2-**A**.

**Table 2:** Relevant Inter- and Intraresidue Correlations from ^1^H–^13^C-HMBC and ^1^H–^1^H NOESY

correlations from anomeric atom		
	HMBC	NOEs
H1, **A**	C2 of **C**/**D**, C3 of **A**, C5 of **A**	H2 of **D**, H2 of **A**
H1, **B**	C3 of **F**, C3 of **B**, C5 of **B**	H3 of **F**, H2 of **B**
H1, **C**	C6 of **C/D**, C5 of **C**	H6 of **CD**, H2 of **C**
H1, **D**	C6 of **C/D**, C5 of **D**	H6 of **C/D**, H2 of **D**
H1, **E**	C2 of **A**, C3 of **E**, C5 of **E**	H2 of **A**, H2 of **E**
H1, **F**	C2 of **A**	H2 of **A**, H2 of **F**
C1, **G**	H6 of **C/D/G**	
H1, **G**		H6 of **G**, H2 of **G**
H1, **I**		H2 of **A**, H2 of **I**

Furthermore, in the DEPT-HSQC experiment, the correlation of the anomeric proton signal at δ 5.34 with a carbon signal at δ 97.5 ppm is consistent with the phosphorylated mannose units.

The occurrence of a phosphodiester linkage was confirmed by the ^1^H–^31^P HMBC experiment, due to the cross-peak between H1-**H** at δ 5.34 ppm and the phosphate signal at δ −1.93 ppm (Figure S8). The latter showed an additional correlation with a proton signal at δ 4.01 ppm (Scheme 1), which was connected in the ^1^H–^13^C DEPT-HSQC experiment with a C2 downfield-shifted carbon at δ 79.8 ppm. All of these cross-peaks suggested a phosphodiester linkage between residue **H** and the 2-substituted mannose of the arms.

All of these data indicated for the Mannan_P.arc_ polysaccharide a backbone of →)6-α-Man-(1→ units), highly branched at position O-2 (Scheme 1). The arms are constituted by oligosaccharides containing only mannose residues substituted at positions O-2 or O-3, ending with mannose or glucose (12%).

**Figure 1:**
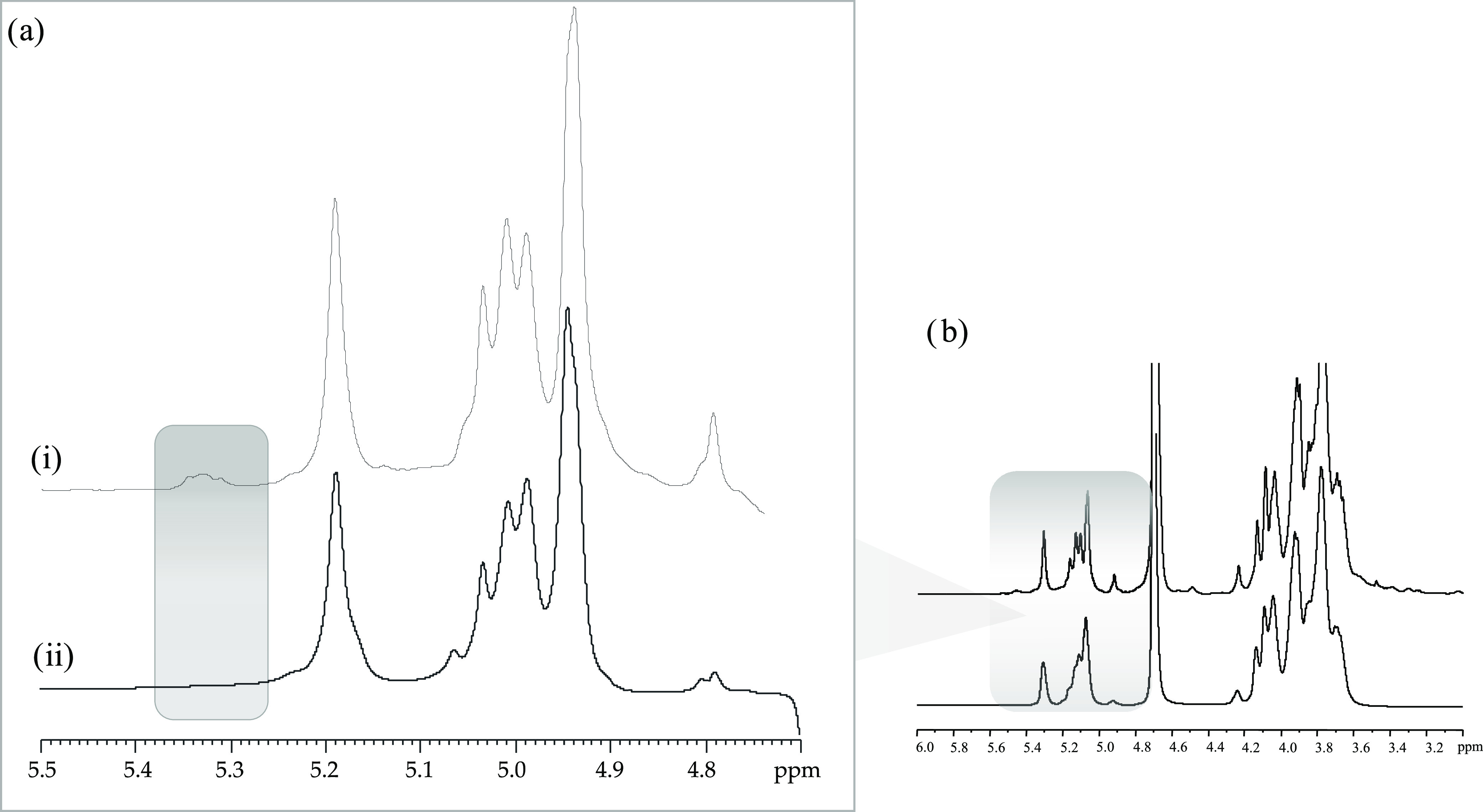
(a) Anomeric regions of ^1^H NMR spectra of (i) Mannan_P.arc_ and (ii) Mannan_Yeast_ and (b) full ^1^H NMR spectra. The spectra were recorded in D_2_O at 298 K and 600 MHz.

To detect the difference between the structure of Mannan_P.arc_ and of the commercial Mannan_Yeast_, a comparison of ^1^H NMR spectra of the two polymers was performed. The spectra revealed a remarkable difference in the anomeric region (Figure 1).

Indeed, the ^1^H NMR (Figure 1) and ^1^H–^13^C DEPT-HSQC spectra (Figure S9) of commercial Mannan_Yeast_ showed the lack of signal at δ 5.34 ppm, attributable to the phosphorylated mannose residues.^36,38^

#### 3.2.2 Mannan_P.arc_HF_

Mannan_P.arc_ was subjected to acid hydrolysis by hydrofluoric acid (HF) to remove the phosphate groups, obtaining Mannan_P.arc_HF_. This reaction allowed us to compare the shapes of mannans in solution by considering the presence or absence of phosphorylation on the polymers. The occurrence of the reaction was checked by ^1^H (Figure S10) and two-dimensional NMR experiments (Figures S11–S15 and Table S1). The anomeric signals of 1-P mannose units at δ 5.34/97.5 ppm were absent in the ^1^H–^13^C DEPT-HSQC spectrum (Figure S11) and a new anomeric signal appeared at δ 4.86/99.0 ppm. The last was assigned to 6-substituted mannose units since C-6 of these residues were downfield-shifted to a value of δ 66.7 ppm. In addition, the methylation analysis of Mannan_P.arc_HF_ revealed a decreased amount of 2-substituted mannose, confirming that the phosphodiester linkage involved these units in the arms.

### 3.3 Physicochemical Characterization of Mannan_P.arc_, Mannan_Yeast_, and Mannan_P.arc_HF_

Besides the basic structure of the polysaccharides, the inter- and intramolecular forces, such as hydrogen bonds, can remarkably affect their conformation, and consequently, their different activity.

Hence, an in-depth physicochemical characterization of the three polysaccharides, Mannan_P.arc_, Mannan_Yeast_, and Mannan_P.arc_HF_, was carried out.

#### 3.3.1 DLS and SLS

**Figure 2:**
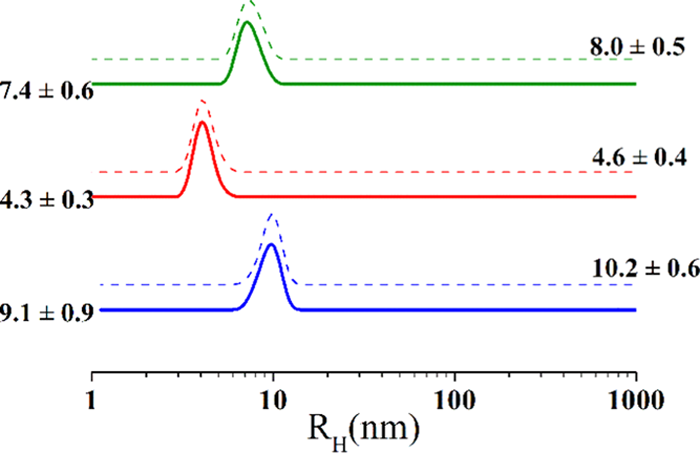
Intensity-weighted hydrodynamic radius distribution measured by DLS of Mannan_P.arc_ (blue), Mannan_Yeast_ (red), and Mannan_P.arc_HF_ (green) at concentrations of 0.2, 0.2, and 0.1 mg mL^–1^ at 25 °C (solid line) and 4 °C (dash line).

Dynamic light scattering performed on Mannan_P.arc_, Mannan_Yeast_, and Mannan_P.arc_HF_ at concentrations of 0.2, 0.2, and 0.1 mg mL^–1^, respectively, and at 4 and 25 °C reveals that all of the systems are characterized by a monomodal distribution independent of the temperature (Figure 2). At 25 °C, Mannan_P.arc_ is characterized by an *R*_H_ value of about 8 nm, Mannan_P.arc_HF_ by a slightly larger value of about 10 nm, while Mannan_Yeast_ is significantly smaller with an *R*_H_ value of about 4 nm. By decreasing the temperature, a slight increase in the value of the hydrodynamic radius is observed for Mannan_P.arc_, as reported in Table 2.

**Figure 3:**
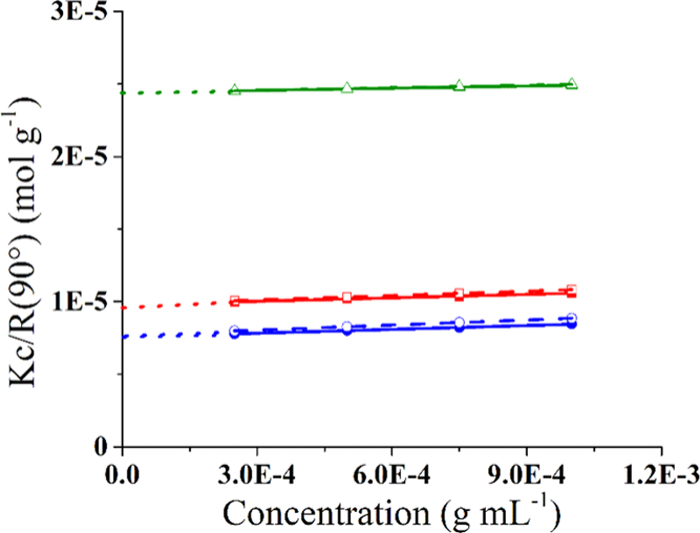
Zimm plot of Mannan_P.arc_ (blue), Mannan_Yeast_ (green), and Mannan_P.arc_HF_ (red) at 25 °C (solid line) and 4 °C (dash line).

Hydrodynamic radius values suggest the presence of single molecules in nonaggregated state, allowing static light scattering (SLS) to be adopted to establish the molecular weight and the second virial coefficient for each of the three polysaccharides. These parameters were determined through a Zimm plot analysis at 4 and 25 °C,^39^ by plotting *Kc*/*R*_θ_ vs polysaccharide concentration (Figure 3).^40^

The mass-averaged molecular weights obtained for Mannan_P.arc_, Mannan_Yeast_, and Mannan_P.arc_HF_ are 13.0 ± 0.9 × 10^4^, 4.1 ± 0.4 × 10^4^, and 10.2 ± 0.8 × 10^4^ Da, respectively (Table 3), with Mannan_Yeast_ presenting the smaller value, in accordance with its smaller dimension. Moreover, for Mannan_Yeast_, the molecular weight is in good agreement with the literature data.^41^

**Table 3:** Main Parameters of Polysaccharides Studied

samples	*R*_H_ (nm) (*T* = 25 °C)	*R*_H_ (nm) (*T* = 4 °C)	*B*_c_ 10^4^ (mol mL g^–2^) (*T* = 25 °C)	*B*_c_ 10^4^ (mol mL g^–2^) (*T* = 4 °C)	*M*_w_ 10^4^ (Da)
Mannan_P.arc_	9.1 ± 0.9	10.2 ± 0.6	4.4 ± 0.3	5.8 ± 0.4	13.0 ± 0.9
Mannan_Yeast_	4.3 ± 0.3	4.6 ± 0.4	2.5 ± 0.2	2.9 ± 0.3	4.1 ± 0.4
Mannan_P.arc_HF_	7.4 ± 0.6	8.0 ± 0.5	4.0 ± 0.2	5.1 ± 0.4	10.2 ± 0.8

From Zimm plot analysis, the second virial coefficients (*B*) of Mannan_P.arc_, Mannan_Yeast_, and Mannan_P.arc_HF_ were determined at both 4 and 25 °C (Table 3). In general, if the second virial coefficient displays positive values, a good solvent condition is suggested, i.e., macromolecular–solvent interactions are favored, whereas negative values indicate a bad solvent condition.^40,42^ In dilute solutions, the polymer conformation, and consequently the dimension of the coil it forms, depends on the interaction between the polymer and the solvent.^43^ In the present case, all of the three polysaccharides exhibit positive values of the second virial coefficient, which in general indicate an efficient water hydration, as already observed for several glycan macromolecules.^44^ The second virial coefficient in the case of the Mannan_Yeast_ is significantly lower and does not change with the temperature with respect to the other two polysaccharides. On the contrary, Mannan_P.arc_ is characterized by the highest value of the second virial coefficient, as well as the largest increase with decreasing temperature. This finding reflects the increase of the hydrodynamic radius of Mannan_P.arc_, which at 4 °C is about 1 nm larger than at 25 °C, which in turn allows for better hydration of the molecule.

#### 3.3.2 Surface Tension Titration

To further investigate the hydrophilic character of the three polysaccharides, surface tension titration was performed at 25 °C up to a polysaccharide concentration of 0.1 mg mL^–1^ (Figure S16). Interestingly, the presence of Mannan_Yeast_ does not affect the surface tension of water. On the contrary, both Mannan_P.arc_ and Mannan_P.arc_HF_ cause a sensible increase of the surface tension, with a ratio *R*_γ_ = γ_mannan_/γ_0_ of about 1.02, an increase of surface tension such as that observed for a NaCl 1 mol L^–1^ water solution.^30^ This result suggests that Mannan_P.arc_ and Mannan_P.arc_HF_ have a marked hydrophilic character.

#### 3.3.3 Circular Dichroism

Insights into the local conformation of the three polysaccharides Mannan_P.arc_, Mannan_Yeast_, and Mannan_P.arc_HF_ in solution were obtained by means of UV circular dichroism. Samples at 0.5 mg mL^–1^ in water were analyzed in the far-UV region at three different temperatures, 4 °C (growth temperature of *P. arcticus*), 20 °C (room temperature), and 37 °C (growth temperature of *S. cerevisiae*).

**Figure 4:**
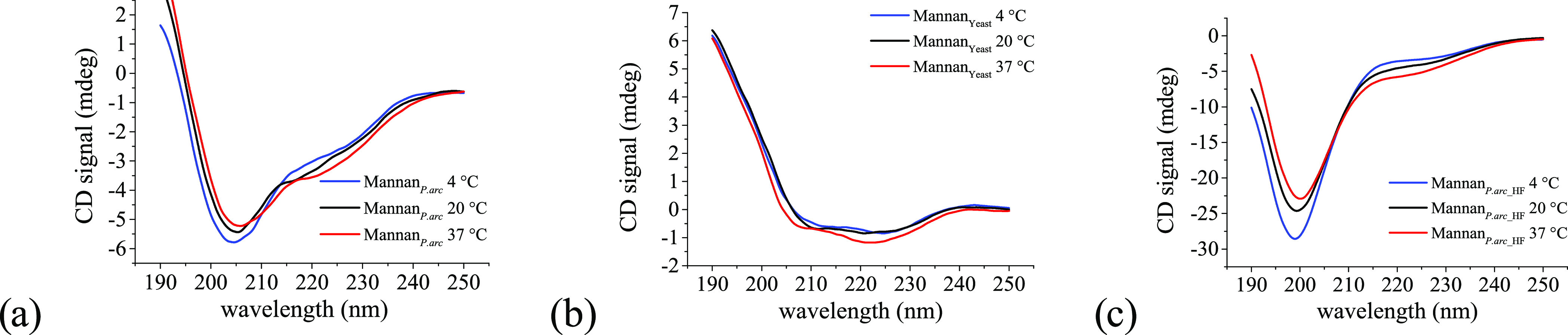
CD profiles of (a) Mannan_P.arc_, (b) Mannan_Yeast_, and (c) Mannan_P.arc_HF_ at 4, 20, and 37 °C.

CD spectra are reported in Figure 4a–c. As clearly emerges from the analysis of Figure 4a–c, the three samples present very different CD spectra, for both shape and intensity (see also Figure S17).

Mannan_P.arc_ is characterized by medium-intensity spectra with a deep minimum at 205 nm, a shoulder around 215–220 nm, and a maximum below 190 nm. Such signals may be indicative of a helical conformation, even if they cannot be associated straightforwardly with a specific kind of helix. Indeed, they are not typical of α-helices in proteins, for which two minima with comparable intensities at 222 and 208 nm are expected. On the other hand, the presence of two minima with different intensities, and in particular, one at 208 nm deeper than that at 222 nm was reported in the case of 3_10_ helices, in particular, a ratio [θ]_222_/[θ]_208_ ≈ 0.4 was taken as an indication of the presence of 3_10_ helices.^45^ In our case, this ratio is about 0.5, but the shifted position of the minima, i.e., 205 nm and 215–220 nm, seems in contrast to this possibility. In the case of proteins, a minimum ranging between 195 and 205 nm is often reported as a spectral feature of polyprII helix, but in our case, the lack of the maximum at 220 nm, the other signature feature of this secondary structure,^46^ seems to point against such a conformation. A deep minimum ranging between 205 and 210 nm has been reported as a signature spectral feature of collagen fibrils, a peculiar supramolecular architecture formed by the triple helix of collagen.^47^

Notably, CD spectra with a marked minimum ranging between 205 and 215 nm have been recorded for β-peptides with different helical conformations. β-Peptides are composed of β amino acids, having an additional carbon atom in the backbone of each residue. They have higher conformational flexibility than α amino acids and may have access to additional secondary structures.^48^ The presence of an extra carbon atom, as well as the possible introduction of constraints like cyclic ring systems could make β-amino acids look more like sugars than α amino acids. CD spectra of Mannan_P.arc_ can indicate the presence of helical structures usually formed by β-peptides, such as the 14-helix. In this respect, it is interesting to note that a 14-helix-bundle formed by a β-peptide has a CD spectrum almost identical to that of our polysaccharide. Therefore, we can infer that Mannan_P.arc_ adopts a local helical conformation, but its structural features cannot be univocally defined. Moreover, inter- and intramolecular interactions between different helices are likely formed determining modification of the spectra with respect to those of known secondary structures.

In the case of Mannan_Yeast_, we observe much less intense minima than those of Mannan_P.arc_ that are positioned at 222 and 208 nm and a maximum below 190 nm. Signals at these wavelengths are usually associated with α-helical conformations, as said before. However, the very low intensity of the minima points toward a very low degree of structuration. Finally, spectra of Mannan_P.arc_HF_ have a single minimum centered at 200 nm that is much more intense than minima in the spectra of the other polysaccharides, typical features of disordered random coil conformations. It clearly emerges that dephosphorylation treatment completely changes spectral features of mannan from *P. arcticus*, likely breaking hydrogen-bonding interactions giving rise to the helical conformation.

Finally, for what concerns the effect of temperature, it is worth noting that the intensity of spectra, that is associated with the degree of structuration, decreases with increasing temperature for Mannan_P.arc_ and Mannan_P.arc_HF_, whereas it increases with increasing temperature for Mannan_Yeast_, in agreement with the different origin of the polysaccharides.

#### 3.3.4 DLS and Cryo-TEM

**Figure 5:**
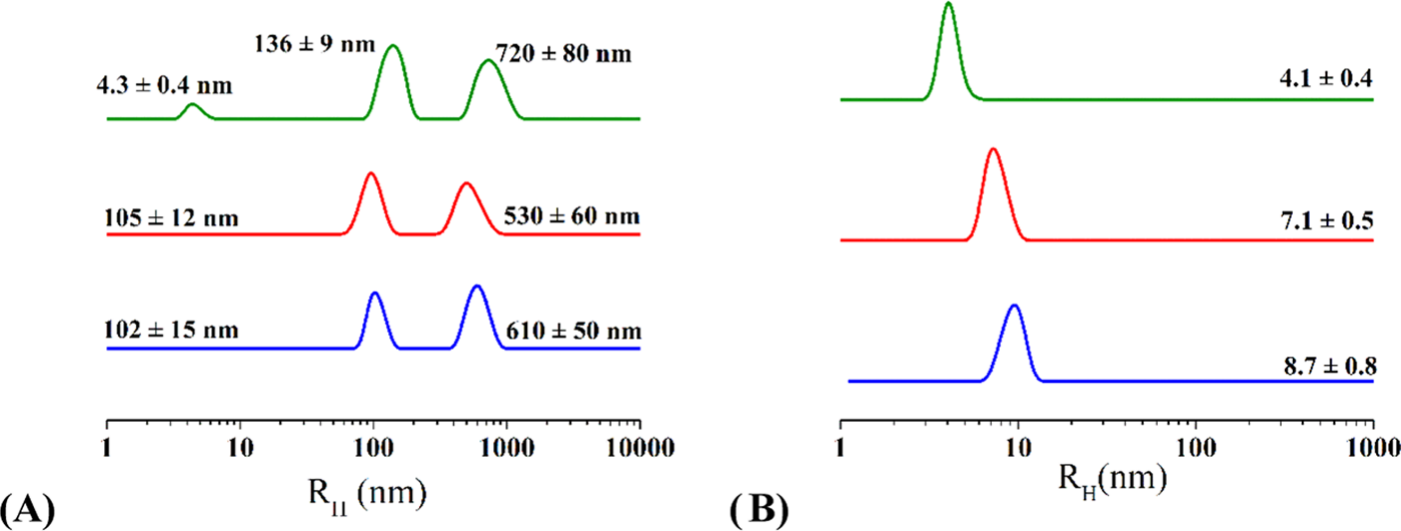
Intensity-weighted (A) and number-weighted (B) hydrodynamic radius distribution measured by DLS of Mannan_P.arc_ (blue), Mannan_Yeast_ (green), and Mannan_P.arc_HF_ (red) at a concentration of ∼1 mg mL^–1^ at 25 °C.

The structural features of the three polysaccharides have been studied also at concentrations 1 order of magnitude higher than those analyzed so far. Samples at 6.3, 6.0, and 5.6 mg mL^–1^ for Mannan_P.arc_, Mannan_Yeast_, and Mannan_P.arc_HF,_ respectively, were analyzed by means of DLS. At these high concentrations, correlation functions particularly of Mannan_P.arc_ do not reach zero values at long times (Figure S18A–C), indicating the presence of suspended very large particles and the beginning of a precipitation process that do not allow determination of DLS profiles. So, samples at about 1 mg mL^–1^ were used for DLS analysis (an example of correlation function for Mannan_P.arc_ sample at this concentration is reported in Figure S18D). DLS profiles of these samples recorded at 25 °C (Figure 5) are showing in all of the cases a main population with significantly higher hydrodynamic radii than observed at a lower concentration, indicating the formation of larger structures by the polysaccharides.

Figure 5A shows that all polysaccharides present two population of aggregates, the size of which ranges for the smaller between 100 and 140 nm and for the larger between 500 and 700 nm. However, while both Mannan_P.arc_ and Mannan_P.arc_HF_ are characterized by the two distribution of aggregates, in the Mannan_Yeast_ solution, there is still a significant number of free chains in solution, as revealed by the distribution centered at about 4 nm. However, DLS is more susceptible to large substances than to smaller ones, with the intensity proportional to the sixth power of radius, so larger substances may hide the presence of smaller ones. To verify this possibility, we performed normalization of the data, allowing the conversion of the intensity-weighted profiles into number-weighted profiles, with intensity proportional to the radius. In this way, we can obtain an indication of the concentration of the different species in the sample. Number-weighted profiles reported in Figure 5B indicate that for all three samples, the presence of free chains in solution is significant.

**Figure 6:**
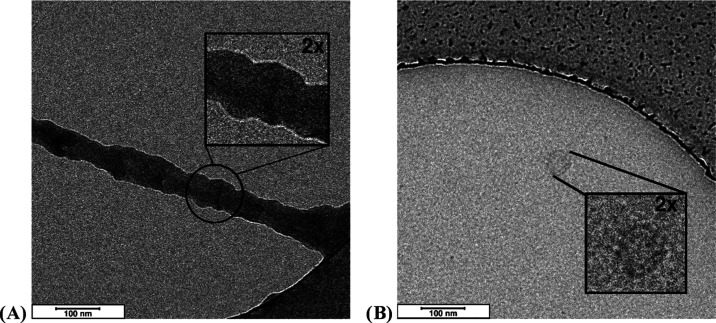
Cryo-TEM images of the Mannan_P.arc_ (A) and Mannan_Yeast_ (B) polysaccharides. Scale bars indicated on both images are 100 nm.

Selected cryo-TEM images collected on Mannan_P.arc_ (Figures 6A and S19) at 6.3 mg mL^–1^ confirm the self-aggregation process evidenced by DLS and indicate the formation of large ribbon structures with length in microns and a diameter of about 40 nm, which were likely responsible for the behavior of the correlation function at long times. Such a structure is evocative of a fibril, similar to that of the collagen, which is indeed characterized by a diameter of the order of tens of nanometers.^49,50^ This finding is quite interesting, also considering indications from CD spectroscopy that could suggest supramolecular aggregation of helical segments into fibril-like arrangements. In the case of Mannan_Yeast_ (Figure 6B), such structures are not present and only coils are evident.

#### 3.3.5 SANS

**Figure 7:**
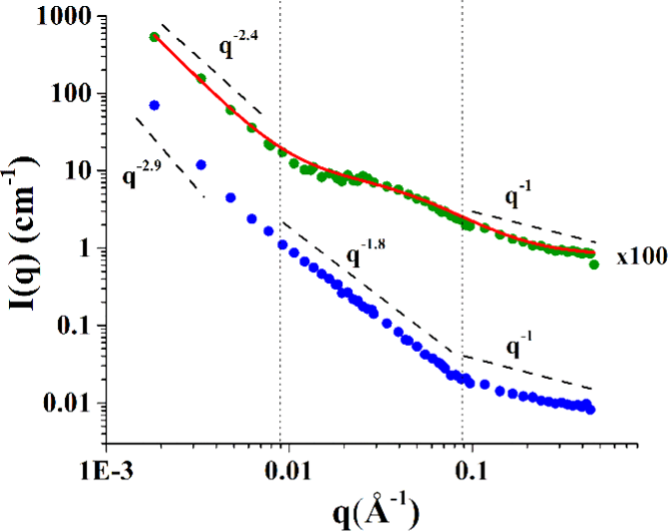
Scattering profile of Mannan_P.arc_ (blue) and Mannan_Yeast_ (green) at ∼1 mg mL^–1^, both experimental points and best-fitting curves.

Finally, the morphology of the polysaccharide aggregates in solution was investigated by small-angle neutron scattering (SANS) (Figure 7) at about 1 mg mL^–1^ concentration, a value significantly lower than the overlapping concentration c* that for polysaccharides and well-hydrated polymers is reported to fall in the 7–10 mg mL^–1^ range.^51−53^ Analysis of SANS profiles at low concentrations comparable to that of DLS, namely, 0.1–0.2 mg mL^–1^, was not possible because of the very large errors, especially in the high-*q* region.

High statistical averaging and short wavelength typical of SANS make direct structural investigations possible on characteristic length scales of a polymer chain, from 1 to 100 nm.

Inspection of Figure 7 shows that in the case of both Mannan_P.arc_ and Mannan_Yeast_, the scattering profile is characterized by three distinct regions: one at small *q*, which corresponds to large scale in direct space and describes the objects or their aggregates as a whole; one at an intermediate *q* range, corresponding to a characteristic length scale in direct space, where one probes the Flory exponent *ν* describing global chain conformation; and finally one at large *q* values, where the local conformation of the chains is probed. The main difference feature between the two systems is in the region around *q* = 0.03 Å^–1^, where for Mannan_Yeast_, a flat region is observed, and for Mannan_P.arc,_ a profile with a slope of −1.8 is visible. At a small *q*, the scattering profile decays with −2.4 and −2.9 slopes for Mannan_Yeast_ and Mannan_P.arc_, respectively. In both cases, this is an indication of the clustering phenomena of the chains. At the higher *q* range, both profiles present a power law with a slope of about −1. In this region, the *q*^–1^ scattering intensity decay indicates that on a local scale, the polysaccharide chain has a rigid-rod behavior, which can be also confirmed by the analysis of the standard Kratky plot, reporting *q*^2^*I(*q*) vs *q* (Figure S20). It is to note that small deviations from the −1 scaling in this region are a likely result of noise in the data and/or background subtraction; indeed, the order of magnitude of the *I*(*q*) values in this range of *q* makes them very sensitive to data processing.

The most interesting region is the intermediate *q* one, where the two polysaccharides have very different behaviors: while the Mannan_Yeast_ profile presents a shoulder that is probably due to a lack of an efficient hydration of chain, as SANS investigation on similar system suggests,^54^ in the case of Mannan_P.arc_, a slope of −1.8 indicates that the chain is well hydrated, and that on a large scale, it has a flexible behavior. In this case, we can determine the Flory exponent *ν* from *q*^–1/*ν*^ obtaining *ν* = 1/1.8 = 0.56, which is very close to the 3/5 value typical of flexible polymer chains in a good solvent.^55^

In the case of Mannan_Yeast_, the following equation was fitted to the scattering profile using a modified correlation function.
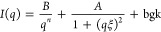
3In the above equation, the first term describes Porod scattering from clusters, while the second term is a Lorentzian function describing scattering from macromolecule chains. The latter accounts for the interaction between the polysaccharide and the solvent. The two multiplicative factors *A* and *B*, the incoherent background bgk, and the exponent *n* are used as fitting parameters. The exponent *n* can be related-to the interaction between the polysaccharide chain and the solvent. The fitting procedure produced values for the correlation length ξ = 25 ± 1 Å and for *n* = 2.4 ± 0.1. The latter value is related inversely to the excluded volume parameter, in particular to the Flory coefficient *ν* = 1/*n* = 0.42. A *v* value between 0.42 and 0.33 suggests a self-attractive interaction within the chain and nonefficient interactions with the solvent.^54^

**Figure 8:**
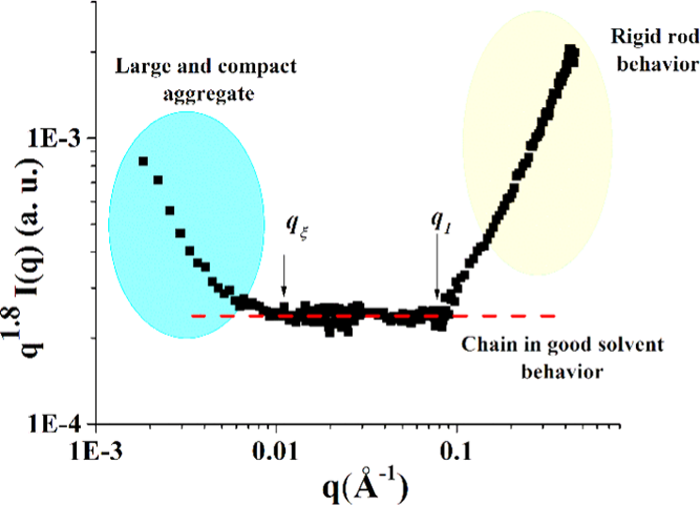
Representation of the SANS data: *q*^1.8^*I*(*q*) vs *q* representation. The onsets in *q* of the different behaviors are discussed in the text.

In the case of Mannan_P.arc_, a characterization was obtained through a *q*^1.8^*I(*q*) vs *q* representation (Figure 8),^55^ which allows us to determine structural local parameters of the chain. The profile in Figure 8 is characterized by a central flat region clearly delimited by two onsets corresponding to the presence of a specific structure in the system at a large scale (*q*_ξ_), which has a typical correlation distance ξ, and to the *q*-limit of the regime, for which the scattering exclusively arises from the stiffness (*q*_1_). From *q*_ξ_ ≈ 0.011 Å^–1^, we can calculate a correlation distance that resulted to be not larger than ∼600 Å by means of ξ = 2π/*q*_ξ_. The upturn below *q*_ξ_ depends on the presence of aggregates and, in this respect, it should be noted that the *q*_ξ_ value could be somewhat affected by the scattering intensity by large aggregates. On the other hand, as said, the *q*^–1^ decay proves that the Mannan_P.arc_ chains have a local stiffness and a rodlike behavior; therefore, they are semiflexible chains. From the *q*_1_ value, it is possible to evaluate the local rigidity, represented by the persistence length *l*_p_, through the relation for polymer chains in good solvent (*ν* = 0.56) *q*_1_ ≈ 3.5/*l*_p_. The calculated *l*_p_ ∼ 40 Å corresponds to 9/10 sugar units, that is, about two repeating units of Mannan_P.arc_.

### 3.4 IRI Activity Assays

**Figure 9:**
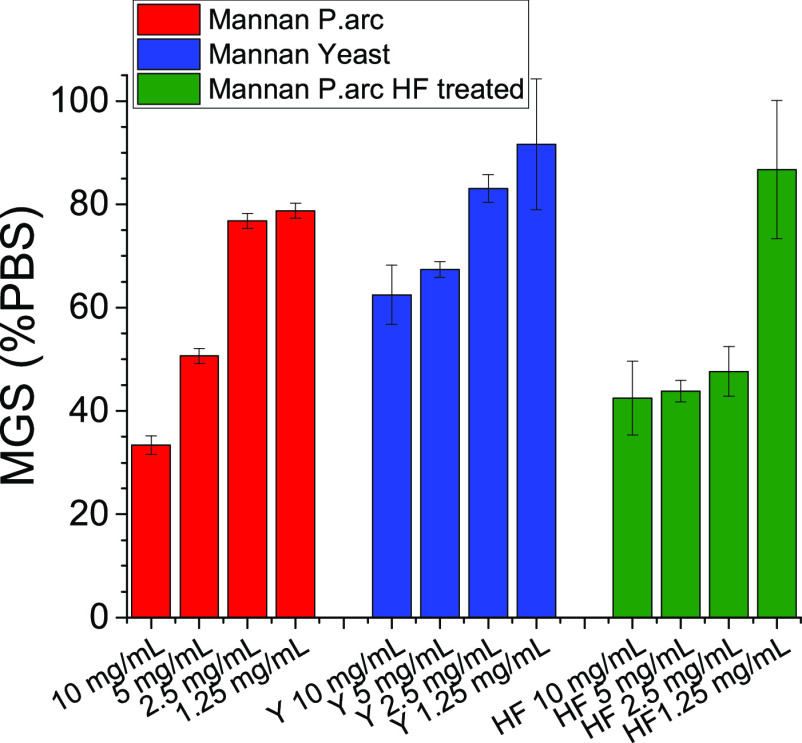
IRI activity of Mannan_P.arc_, Mannan_Yeast_, and Mannan_P.arc_HF_. Mean grain size (MGS) of the ice crystals size is expressed as a percentage of PBS, and smaller MGS values indicate increased IRI activity.

To determine if these polysaccharides had the capacity to modulate ice growth and ice recrystallization inhibition (IRI), assays were undertaken.^56^ IRI was determined by the splat assay, whereby a polynucleated ice wafer was allowed to grow at −8 °C for 30 min, and the relative size of the crystals compared to a PBS control was evaluated. Smaller ice crystals indicate greater ice recrystallization activity. The results highlighted some weak ice recrystallization inhibition activity of the Mannan_P.arc_ polymer, which was slightly higher than the IRI activity of Mannan_Yeast_ (Figure 9). It should be noted that this material is significantly less active than potent IRIs such as poly(vinyl alcohol),^56,57^ antifreeze proteins, or recently reported amphiphilic metalohelicies^58^ but more than negative controls such as poly(ethylene glycol). It is important to note that sufficient concentrations of any macromolecule can slow down ice growth and that conducting the assays in the presence of saline (or e.g., sucrose) is essential to remove false positives.^59^

Mannan_P.arc_HF_ was then tested for the IRI activity (Figure 9). Interestingly, no large differences are seen between the two different samples, with both exhibiting weak IRI activity. In the case of the Mannan_P.arc_HF_ sample, activity is retained at a lower concentration (2.5 mg mL^–1^) before being lost completely at 1.25 mg mL^–1^, but this is not a significant difference. The bioassays indicated that the lack of phosphate groups did not substantially affect the activity and do not determine the lack of the activity comparable to that of Mannan_Yeast_.

While the activity for all samples seen in this work is weak, several polysaccharides, such as those based on fucose, are emerging as potent cryoprotectants and hence the study of polysaccharides with any activity may form the basis for discovering new cryoprotectants.^58^ The exact relationship between IRI and cryoprotectant outcomes is also not fully understood, and hence these (or other) extracellular polysaccharides from cold-acclimatized bacteria which do not have significant IRI, may provide protection by another mechanism.

## 4 Discussion

Mannan polysaccharides can be found as extracellular microbial components. They are usually highly branched polymers, characterized by the absence of a defined repeating unit. Therefore, they are mainly investigated for their activity, whereas the deep structural investigation is often overlooked. For example, in *Pseudomonas mutabilis*,^60^ the isolated mannan showed high-viscosity solution, whereas in *Rhodopseudomonas palustris*,^61^ the polysaccharide solutions have been reported to influence the growth of beneficial gut microbiota. Neither these studies nor those reporting the structure and the activities of mannans isolated from fungi have considered the structure/activity relationships. In addition, up to now, only a few papers describing conformational analysis of microbial extracellular polysaccharides are known; therefore, there is a need for bridging this gap.

In this study, we present the structure and conformational analysis of a mannan extracellular polysaccharide purified from the cold-adapted *P. arcticus* 273-4 (Mannan_P.arc_) displaying weak to moderate IRI activity higher than that of *S. cerevisiae* (Mannan_Yeast_). The Mannan_P.arc_ structure is constituted of a backbone of 6-substituted mannose residues, which is highly ramified at C-2, with di- and trisaccharide side chains containing 2- and 3-substituted mannose units, respective. Glucose residues can be found as a terminal unit of some arms. The phosphodiester linkage connects some terminal mannose units to the 2-substituted ones. Our results indicate that this structural feature agreed with previously published data.^12,62^

It is worth noting that *P. arcticus* 273-4 produces both CPS^63^ and medium released polysaccharides, and only the last have found to display moderate IRI activity (this study). To date, the production of mannan polysaccharides is documented for other psychrophiles,^12,64^ even if to our knowledge, no reports about the crucial role of the conformation that directly affects the activity have been found in the literature.

Therefore, we aimed to evaluate several physicochemical properties of the mannans in solution to figure out their possible different shapes. The three polymers were considered, i.e., Mannan_P.arc_, Mannan_Yeast_, and Mannan_P.arc_HF_, and compared. The latter was obtained from Mannan_P.arc_ using hydrofluoric acid, to evaluate the role of the phosphorylated mannose in defining the observed shape.

All of the three molecules showed an efficient hydration, which for Mannan_P.arc_ is more marked and increases with decreasing temperature, as shown by the second virial coefficient values. Moreover, both Mannan_P.arc_ and Mannan_P.arc_HF_ were characterized by a distinct hydrophilicity typically observed in moderate concentrated salt-water solution, as highlighted by surface tension measurements.

At high concentrations, all of the three polysaccharides showed the tendency to form a larger structure, even if a significant concentration of free chain is still present, as clearly indicated by intensity-weighted and number-weighted DLS profiles, respectively. The presence of aggregates in the case of Mannan_P.arc_ and Mannan_Yeast_ is also proved by the SANS profiles at low *q* values. SANS analysis also indicates that both polysaccharides adopt a local rigid structure along the chain. Mannan_P.arc_ is a semiflexible chain characterized by a rigid part of the chain that, according to the calculated persistence length value, is not especially large, as, in line with those of most polysaccharides, extends up to about 9–10 residues of the molecules. According to CD measurements, these rigid-rod regions are likely to adopt a helical conformation and interact by forming hierarchical organization. These helical regions encompass a relatively few residues, presuming a conformation similar to that of helices formed by β-peptides, and it is worth saying that, in contrast to α-helices, these may be stable even when formed by only a few residues.^65^

On the other hand, the organization in hierarchical architectures may stabilize helices that are intrinsically unstable, such as the case of other polysaccharides,^19^ and contribute to the overall conformation of the polysaccharide or to the formation of aggregates. Cryo-TEM images seem to confirm the latter hypothesis, showing the formation of very large ribbon-like structures evocative of fibrils in the case of Mannan_P.arc_. On the other hand, Mannan_Yeast_ seems to be characterized by a very low degree of structuration in α-helical conformation. Finally, CD shows that dephosphorylation destroys the local organization of the polysaccharide chain, with the CD spectra of Mannan_P.arc_HF_ being typical of disordered random coil conformations, by altering intramolecular interactions stabilizing the helical segments or the supramolecular assembly. Indeed, charge modifications can destroy superhelical organization and isolated helices may become unstable and unfold.^19^

Finally, since the IRI assays of Mannan_P.arc_HF_ did not show significant differences with respect to the intact polymer, we concluded that the phosphate groups did not display a relevant role in the bioactivity of the *P. arcticus* polymer. However, the presence of helical domains in Mannan_P.arc_ can be crucial for other activities since these domains are often responsible for interchain associations giving rise to a three-dimensional network with viscoelastic behavior, a gel, by providing noncovalent cross-linking in the junction zones.

Our results may suggest a significant difference between “functional” and “structural” cold adaptation mechanisms that need further investigation: while enzymes and membranes of psychrofilic organisms preserve their functional role at cold temperatures through an either overall or local increase of flexibility and disorder,^66,67^ other macromolecules, such as EPS, may accomplish their protective role through increased rigidity and structuring.

## 5 Conclusions

This study presents a methodology that is not commonly used for establishing a correlation between the structure and the shape of polysaccharides in solution in the absence of a defined oligosaccharidic repeating unit. The approach exploits different physicochemical methods, some of which, like cryo-TEM, have never been used for obtaining such information. We employed this approach to understand the differences among the mannan from the Siberian permafrost *P. arcticus* bacterium, its dephosphorylated derivative, and the mannan from *S. cerevisiae*.

The collected data reveal that the mannan from *P. arcticus* bacterium has an efficient interaction with the water molecules, and it is structurally characterized by a rigid part of the chain, about 9/10 sugar units, i.e., two repeating units of Mannan_P.arc_, that assumes a helical conformation. Dephosphorylation seems to destroy this local organization of the polysaccharide chain. Finally, the mannan from *S. cerevisiae* seems to be characterized by a very low degree of structuration in α-helical conformation. The psychrophilic mannans showed a weak IRI activity if compared with the nonactive mannan’s yeast. Future studies will clarify which structural feature is responsible for the different behavior.

## Supporting Information Available

Experimental details; gel filtration chromatogram; and NMR spectra (PDF)
