# Supplementary material for: Physicochemical Approach to Understanding the Structure, Conformation, and Activity of Mannan Polysaccharides
Source: Biomacromolecules. 2021 Mar 17;22(4):1445–57. doi: 10.1021/acs.biomac.0c01659 (PMC8045027; doi:10.1021/acs.biomac.0c01659)
Supplement: Supplementary file 1 — bm0c01659_si_001.pdf [file bm0c01659_si_001.pdf]

Supporting Information

A Physico-chemical approach to understanding the structure, the conformation, and the activity of mannan polysaccharides.

Angela Casillo,[a] *Antonio Fabozzi,*[a] *Irene Russo Krauss,*[a,b] *Ermenegilda Parrilli,*[a] *Caroline I. Biggs,*[c] *Matthew I. Gibson,*[c,d] *Rosa Lanzetta,*[a] *Marie-Sousai Appavou,*[e] *Aurel Radulescu,*[e,] *Maria L. Tutino,*[a] *Luigi Paduano,*[a,b*] *Maria M. Corsaro*[a*]

[a] Department of Chemical Sciences, University of Naples "Federico II", Complesso Universitario Monte S. Angelo, Via Cintia 4, 80126 Naples, Italy

[b] CSGI - Consorzio per lo Sviluppo dei Sistemi a Grande Interfase, Florence, Italy

[c] Department of Chemistry, University of Warwick, Coventry, CV4 7AL, UK

[d] Warwick Medical School, University of Warwick, Coventry, CV4 7AL, UK

[e] Jülich Centre for Neutron Science, Garching Forschungszentrum, Lichtenbergstrasse 1, D-857478 Garchingbei München, Germany

[*] E-mail address: lpaduano@unina.it (Luigi Paduano); corsaro@unina.it (Maria Michela Corsaro)

Figure S1. Sephacryl S-400 HR gel filtration chromatogram profile of the precipitate from the growth medium from *P.arcticus* 273-4.

Figure S2. ^{1}H-^{13}C DEPT-HSQC spectrum of mannan from *P.arcticus* 273-4 (Mannan$_{\boldsymbol{P.arc}}$). The spectrum was recorded in D_2O at 298 K at 600 MHz.

Figure S3. Relevant section of 2D *F2*-coupled HSQC experiment of Mannan$_{\boldsymbol{P.arc}}$ measured in D_2O at 298 K at 600 MHz.

Figure S4. Expansion of the ^{1}H-^{13}C HMBC spectrum of Mannan$_{\boldsymbol{P.arc}}$. The spectrum was recorded in D_2O at 298 K at 600 MHz.

Figure S5. Expansion of the ^{1}H-^{1}H NOESY spectrum of Mannan$_{P.arc}$ measured at 298 K in D_2O (150 ms as mixing time) at 600 MHz.

Figure S6. Expansion of the^{1}H-^{1}H COSY spectrum of Mannan$_{P.arc}$ measured in D_2O at 298 K at 600 MHz.

Figure S7. Expansion of the ^{1}H-^{1}H TOCSY spectrum of Mannan$_{P.arc}$ measured in D_2O at 298 K at 600 MHz.

Figure S8. Expansion of ^{1}H-^{31}P HMBC of Mannan$_{P.arc}$. The spectrum was recorded in D_2O at 298 K at 400 MHz.

Figure S9. Expansions of the ^{1}H-^{13}C DEPT-HSQC spectra of mannans from a) *P.arcticus* 273-4 (Mannan$_{\boldsymbol{P.arc}}$), and b) *S. cerevisiae*. The spectra were recorded in D_2O at 298 K at 600 MHz.

Figure S10. ^{1}H NMR spectrum of the from *P. arcticus* (Mannan$_{P.arc_\mathrm{HF}}$). The spectrum was recorded in D_2O at 298 K at 600 MHz.

Figure S11. ^{1}H-^{13}C DEPT-HSQC spectrum of Mannan$_{P.arc_\mathrm{HF}}$. The spectrum was recorded in D_2O at 298 K at 600 MHz.

Figure S12. Expansion of the ^{1}H-^{1}H COSY spectrum of Mannan$_{P.arc_\mathrm{HF}}$ measured in D_2O at 298 K at 600 MHz.

Figure S13. Expansion of the ^{1}H-^{1}H TOCSY spectrum of Mannan$_{P.arc_\mathrm{HF}}$ measured in D_2O at 298 K at 600 MHz.

Figure S14. Expansion of the ^{1}H-^{1}H NOESY spectrum of Mannan$_{P.arc_\mathrm{HF}}$ measured at 298 K in D_2O (150 ms as mixing time) at 600 MHz.

Figure S15. Expansion of the ^{1}H-^{13}C HMBC spectrum of Mannan$_{\boldsymbol{P.arc}_\mathrm{HF}}$. The spectrum was recorded in D_2O at 298 K at 600 MHz.

Figure S16. Surface tension as a function of Mannan$_{P.arc}$ (blue), Mannan$_{\mathrm{Yeast},}$ (green) and Mannan$_{P.arc_\mathrm{HF}}$ (red) concentration at 25.0 °C.

Figure S17. Overlapped CD spectra of Mannan$_{P.arc}$ (blue), Mannan$_{\mathrm{Yeast}}$ (green) and Mannan$_{P.arc_\mathrm{HF}}$ (red) at 20 °C.

Figure S18. Normalized correlation functions for Mannan$_{P.arc}$ (A), Mannan$_{\mathrm{Yeast}}$ (B) and Mannan$_{P.arc_\mathrm{HF}}$ (C) at 6.3, 6.0 and 5.6 mg mL^{-1}, respectively, and for Mannan$_{P.arc}$ at 1 mg mL^{-1} (D).

Figure S19. Cryo-TEM images collected on Mannan$_{P.arc}$.

Figure S20. Kratky plot of SANS data for Mannan$_{\mathrm{Yeast}}$.

Table S1. ^{1}H and ^{13}C NMR assignments of Mannan$_{P.arc_\mathrm{HF}}$. Spectra were recorded in D_2O at 298 K at 600 MHz using acetone as external standard (δ_H/δ_C 2.25/ 31.45 ppm).

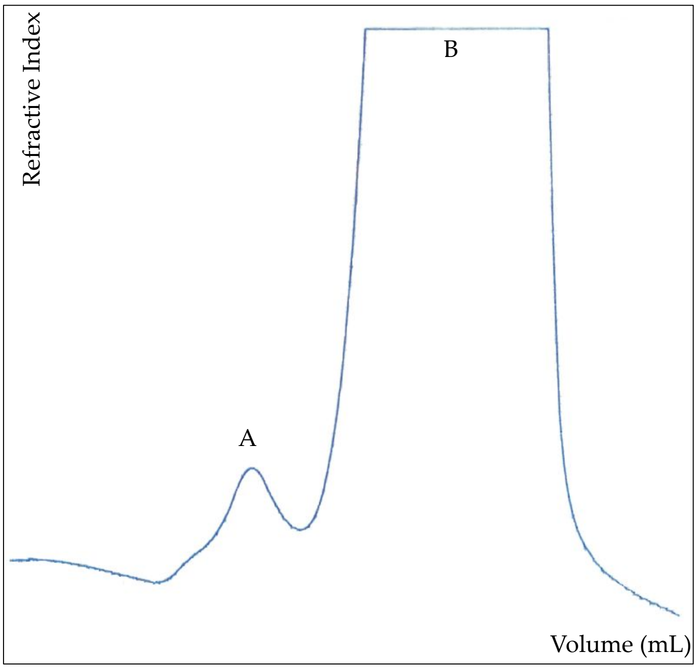

Figure S1. Sephacryl S-400 HR gel filtration chromatogram profile of the precipitate from the growth medium from *P.arcticus* 273-4.

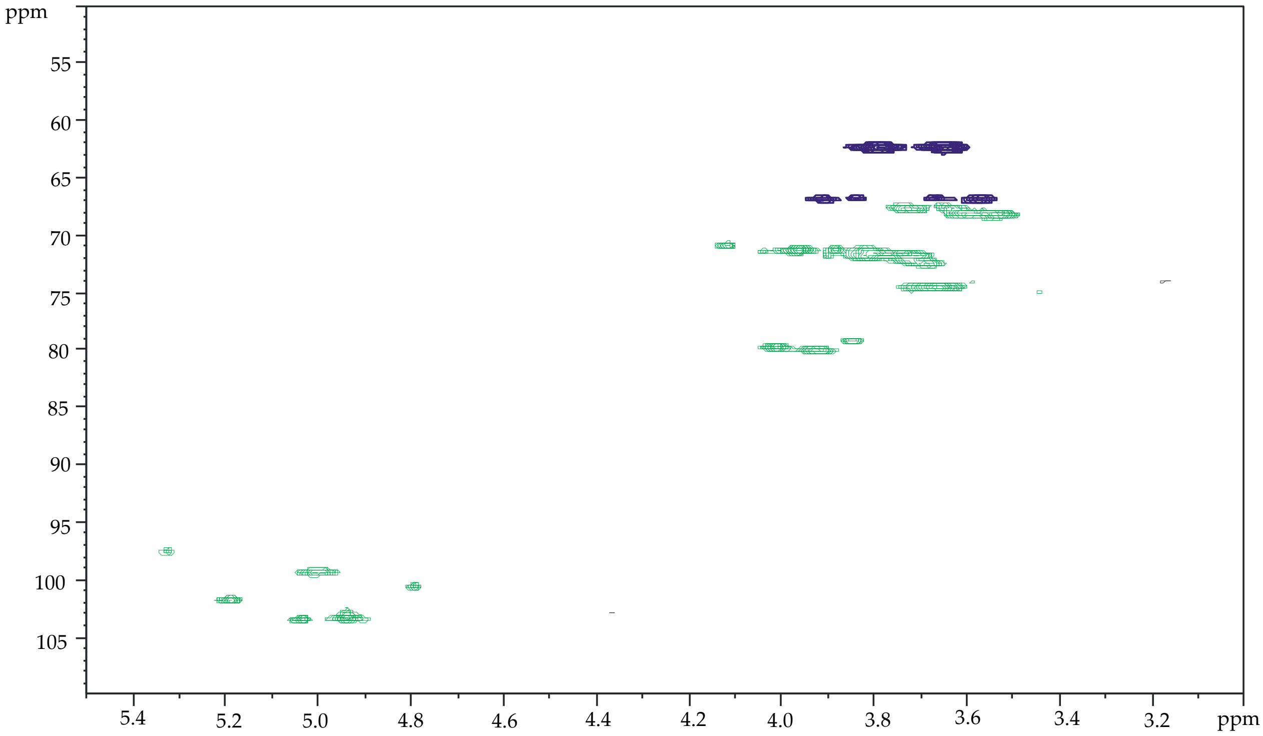

Figure S2. ^{1}H-^{13}C DEPT-HSQC- spectrum of mannan from *P.arcticus* 273-4 (Mannan$_{\mathbf{\textit{P.arc}}}$). The spectrum was recorded in D_2O at 298 K at 600 MHz.

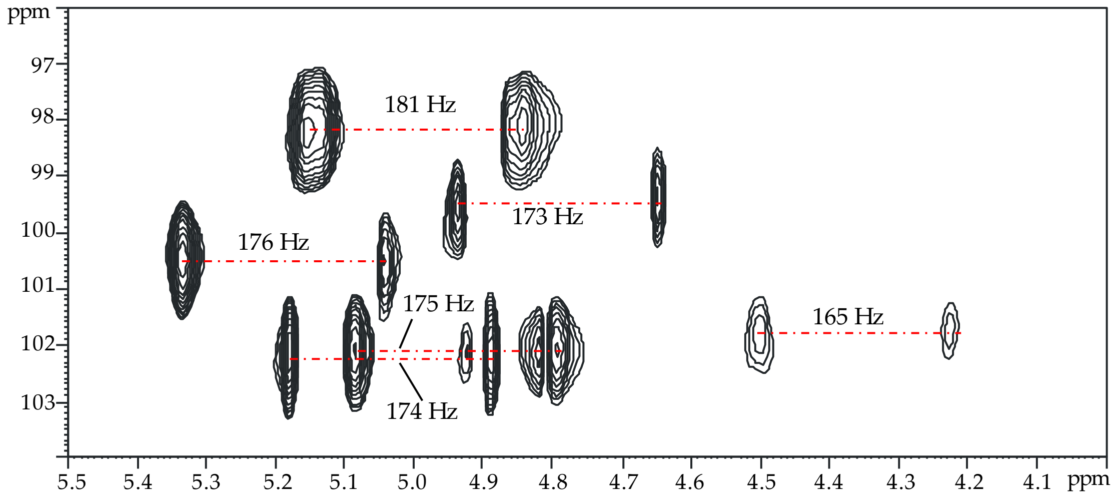

Figure S3. Relevant section of 2D *F2*-coupled HSQC experiment of Mannan$_{\boldsymbol{P.arc}}$ measured in D_2O at 298 K at 600 MHz.

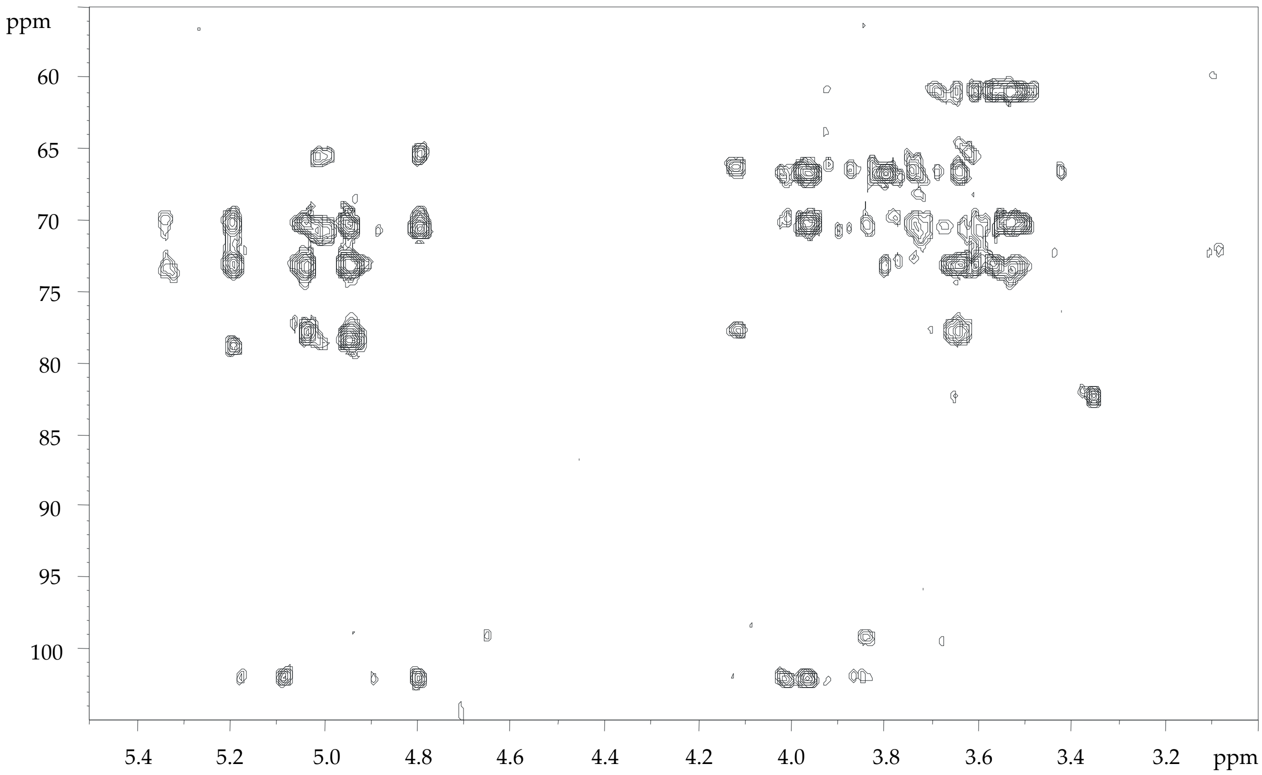

Figure S4. Expansion of the ^{1}H-^{13}C HMBC spectrum of Mannan$_{\boldsymbol{P.arc}}$. The spectrum was recorded in D_2O at 298 K at 600 MHz.

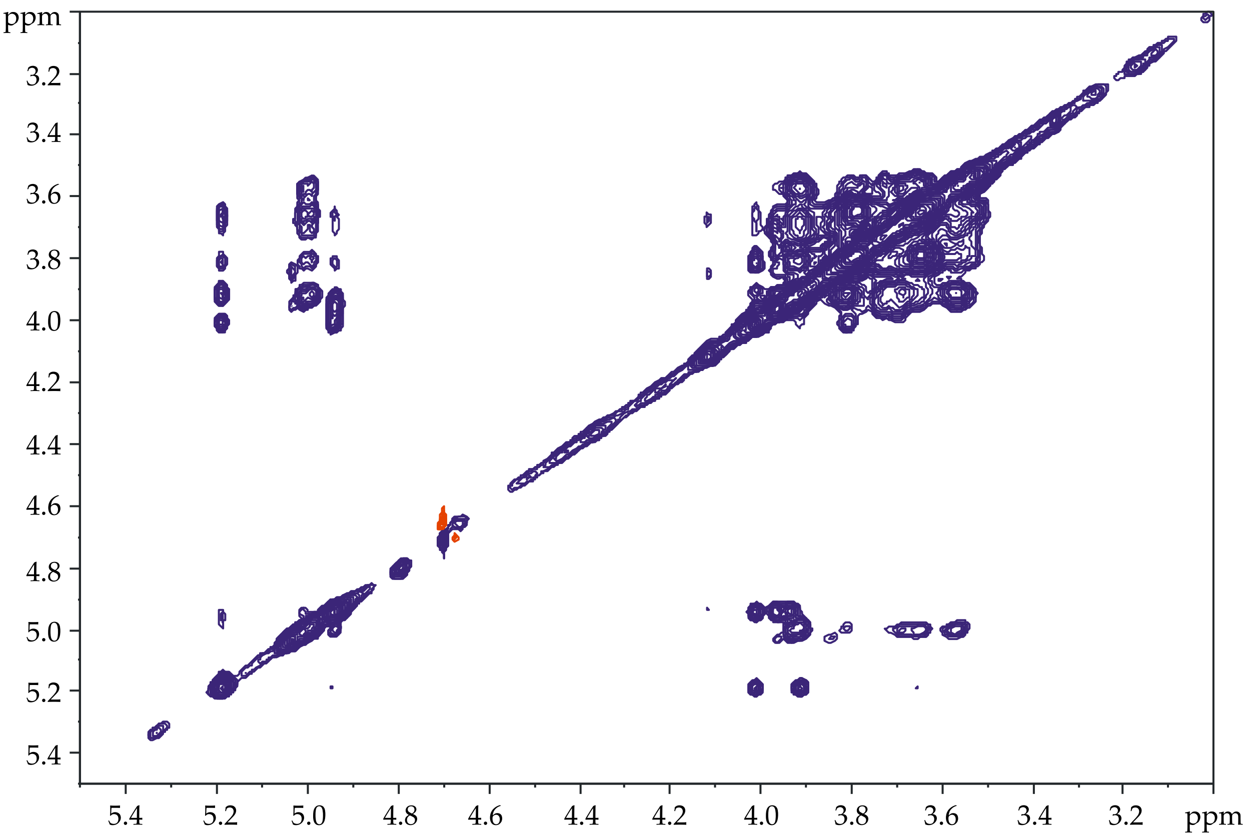

Figure S5. Expansion of the ^{1}H-^{1}H NOESY spectrum of Mannan$_{P.arc}$ measured at 298 K in D_2O (150 ms as mixing time) at 600 MHz.

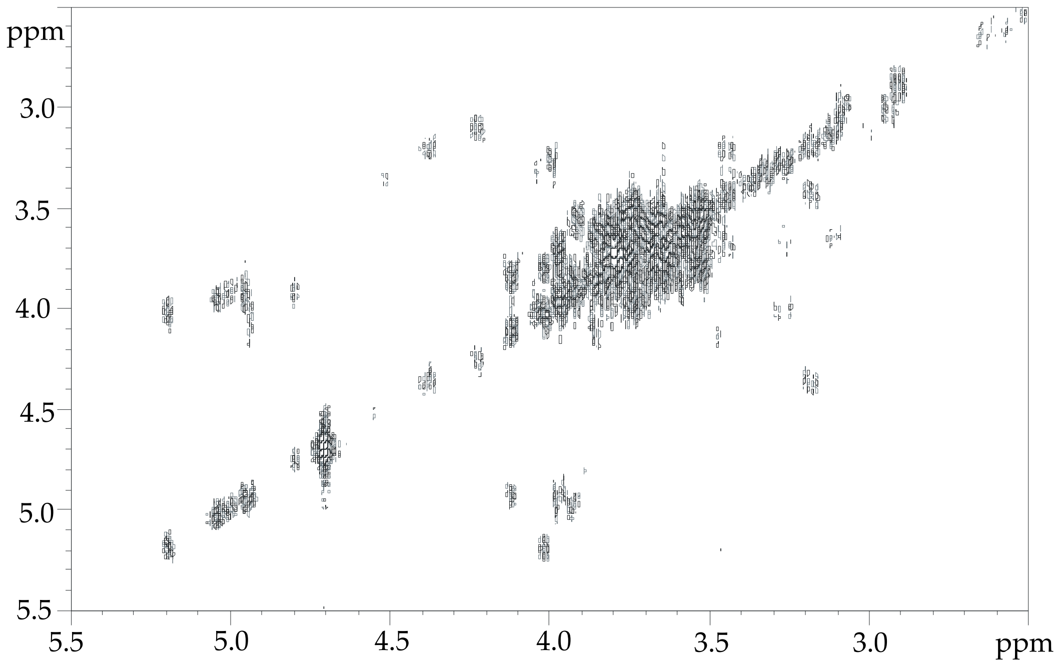

Figure S6. Expansion of the^{1}H-^{1}H COSY spectrum of Mannan$_{P.arc}$ measured in D_2O at 298 K at 600 MHz.

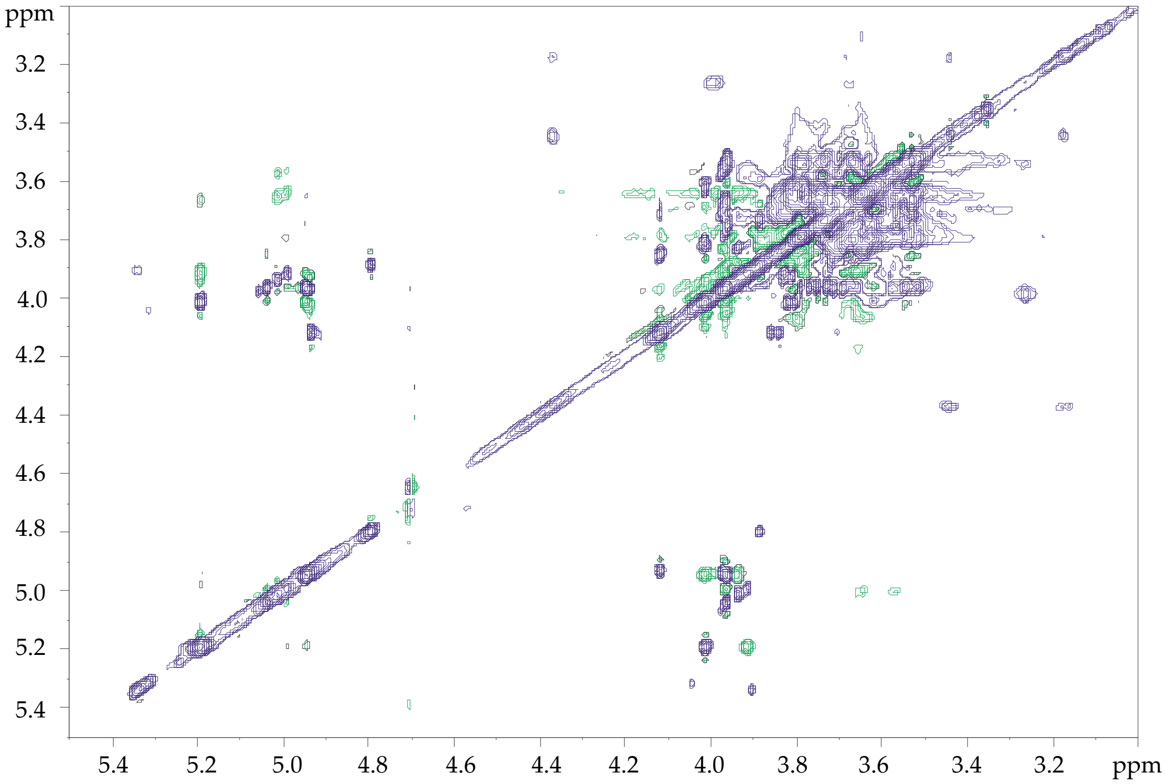

Figure S7. Expansion of the ^1H-^1H TOCSY spectrum of Mannan$_{P.arc}$ measured in D_2O at 298 K at 600 MHz.

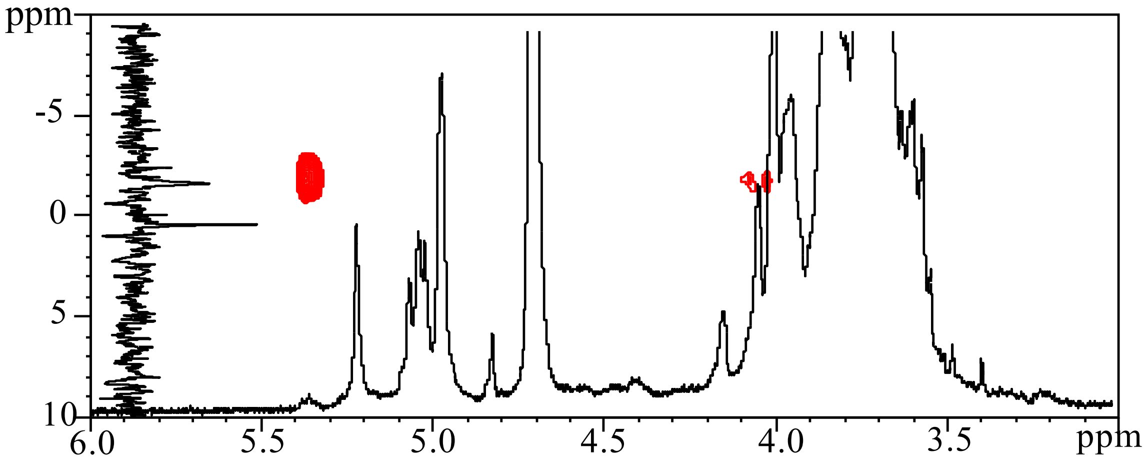

Figure S8. Expansion of ^1H-^{31}P HMBC of Mannan$_{P.arc}$. The spectrum was recorded in D_2O at 298 K at 400 MHz.

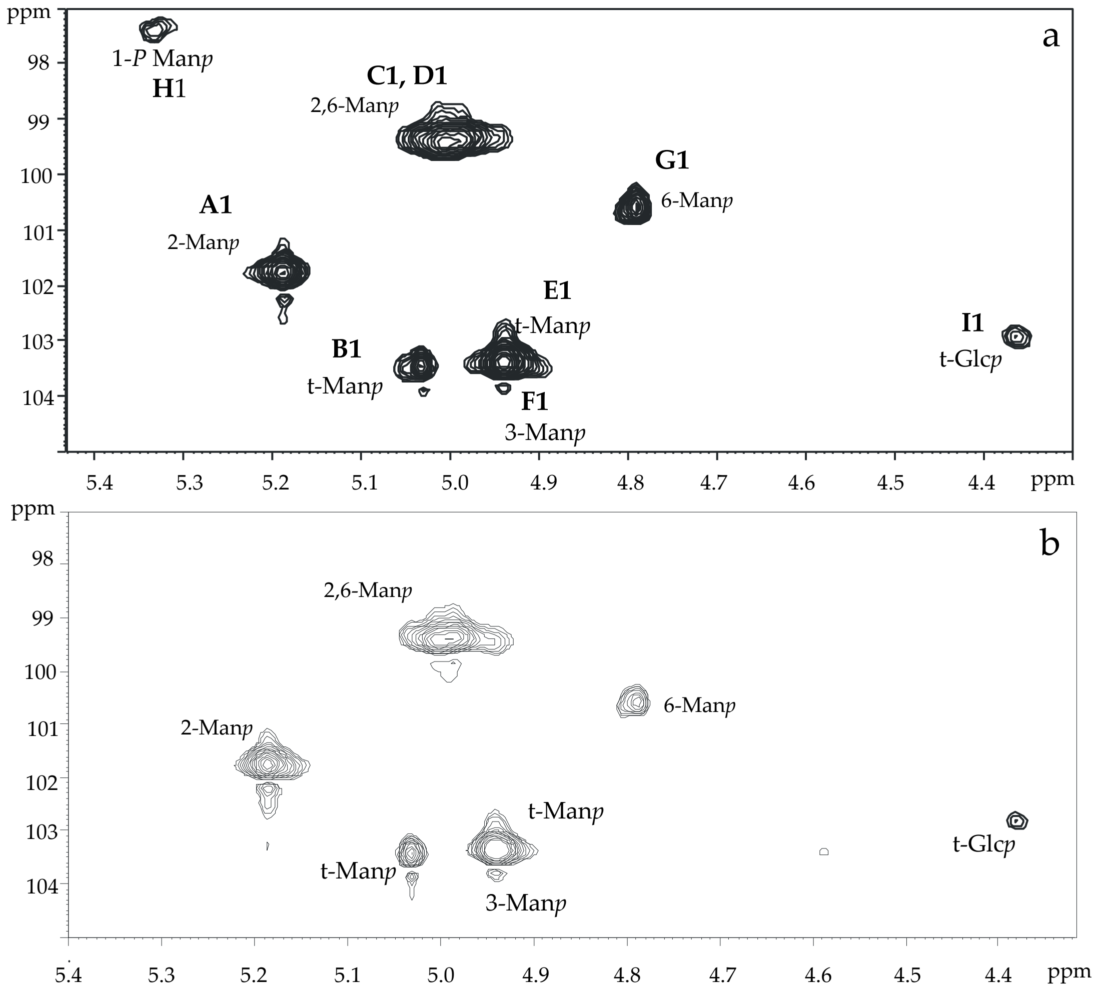

Figure S9. Expansions of the ^{1}H-^{13}C DEPT-HSQC spectra of mannans from a) *P.arcticus* 273-4 (Mannan$_{P.arc}$), and b) *S. cerevisiae*. The spectra were recorded in D_2O at 298 K at 600 MHz.

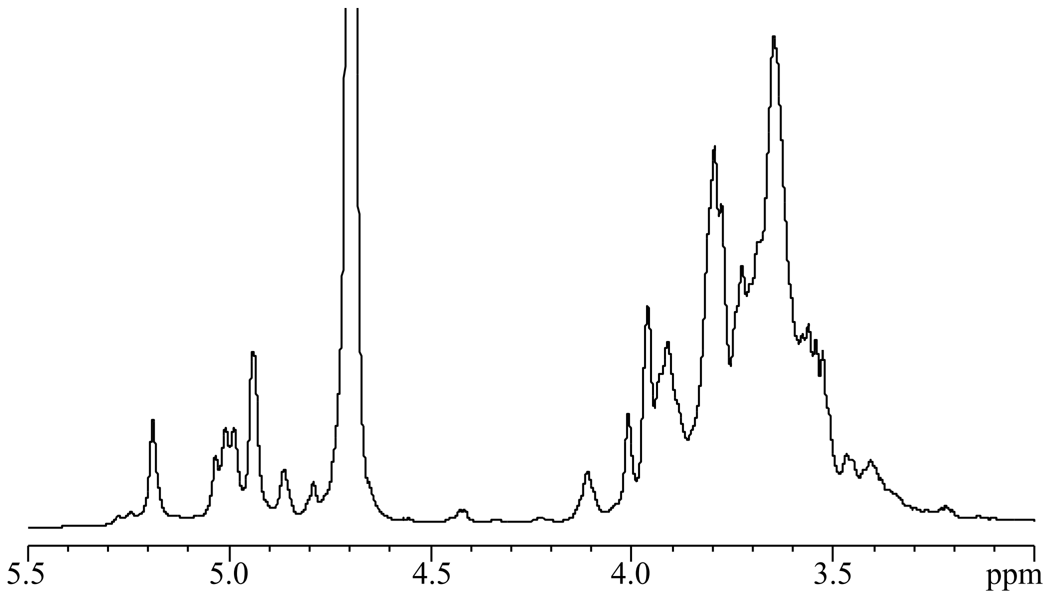

Figure S10. ^{1}H NMR spectrum of the from *P. arcticus* (Mannan$_{P.arc_HF}$). The spectrum was recorded in D_2O at 298 K at 600 MHz.

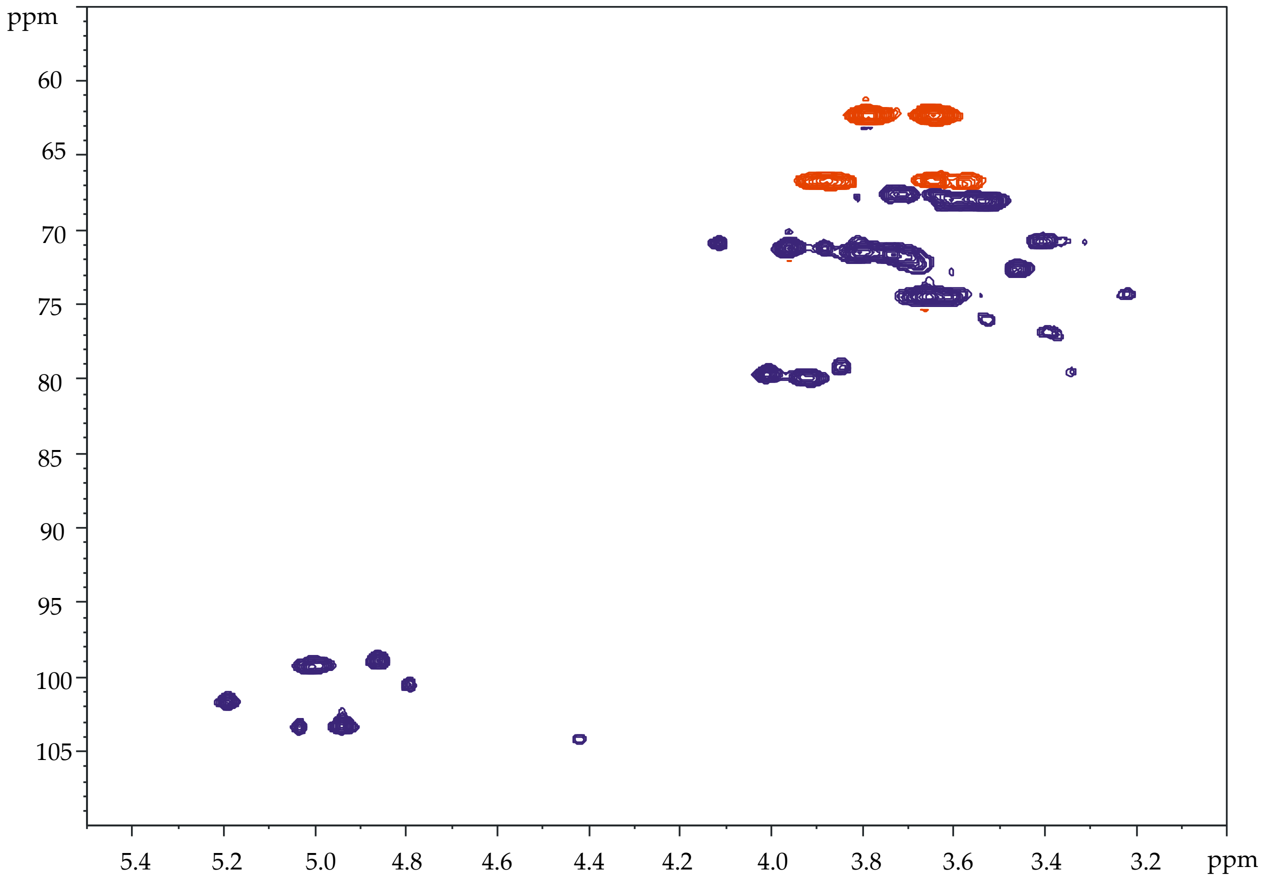

Figure S11. ^{1}H-^{13}C DEPT-HSQC spectrum of Mannan$_{P.arc_HF}$. The spectrum was recorded in D_2O at 298 K at 600 MHz.

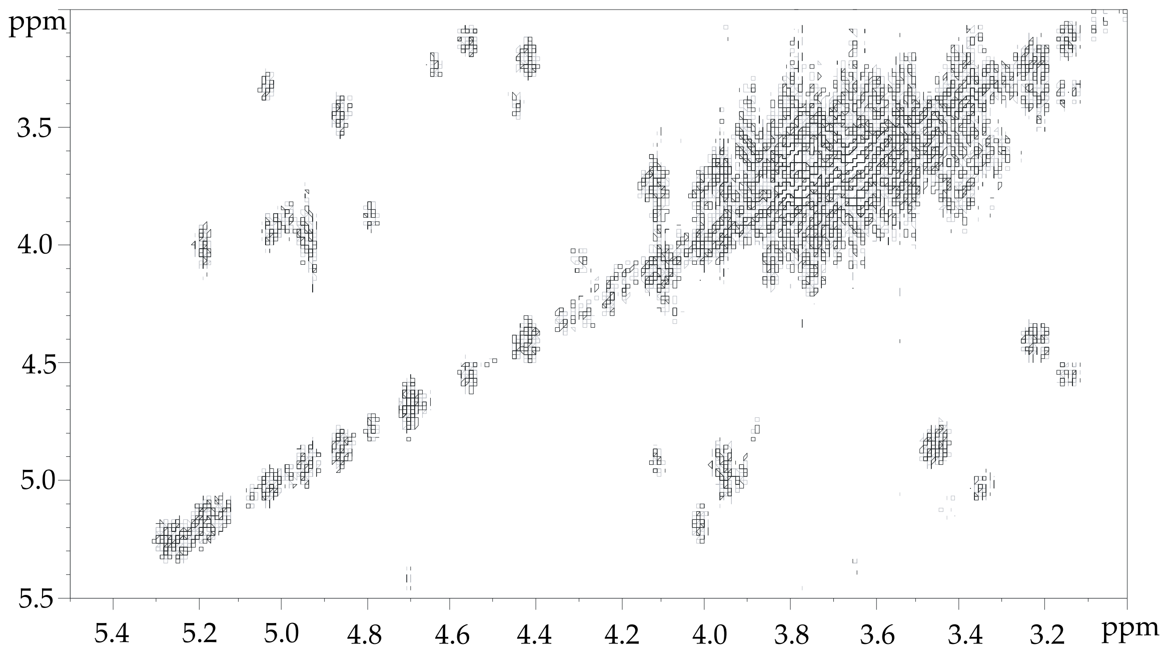

Figure S12. Expansion of the ^{1}H-^{1}H COSY spectrum of Mannan$_{P.arc_HF}$ measured in D_2O at 298 K at 600 MHz.

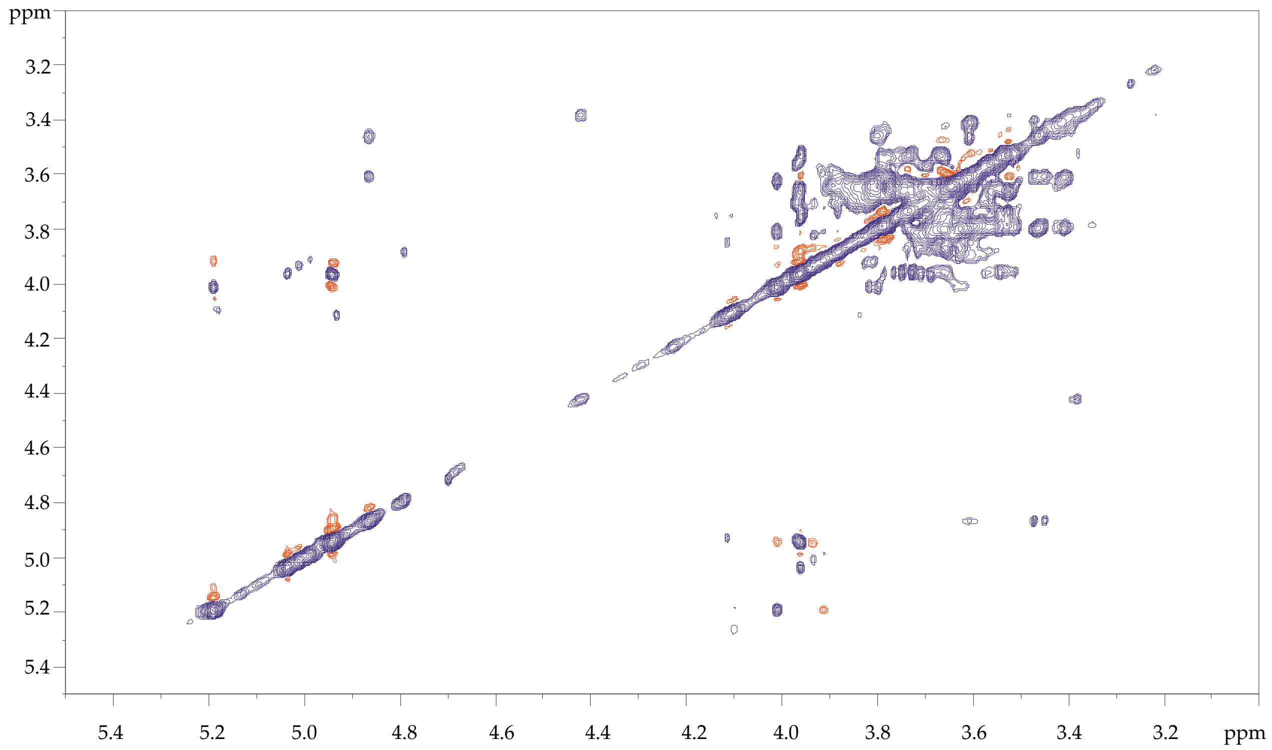

Figure S13. Expansion of the ^{1}H-^{1}H TOCSY spectrum of Mannan$_{P.arc_HF}$ measured in D_2O at 298 K at 600 MHz.

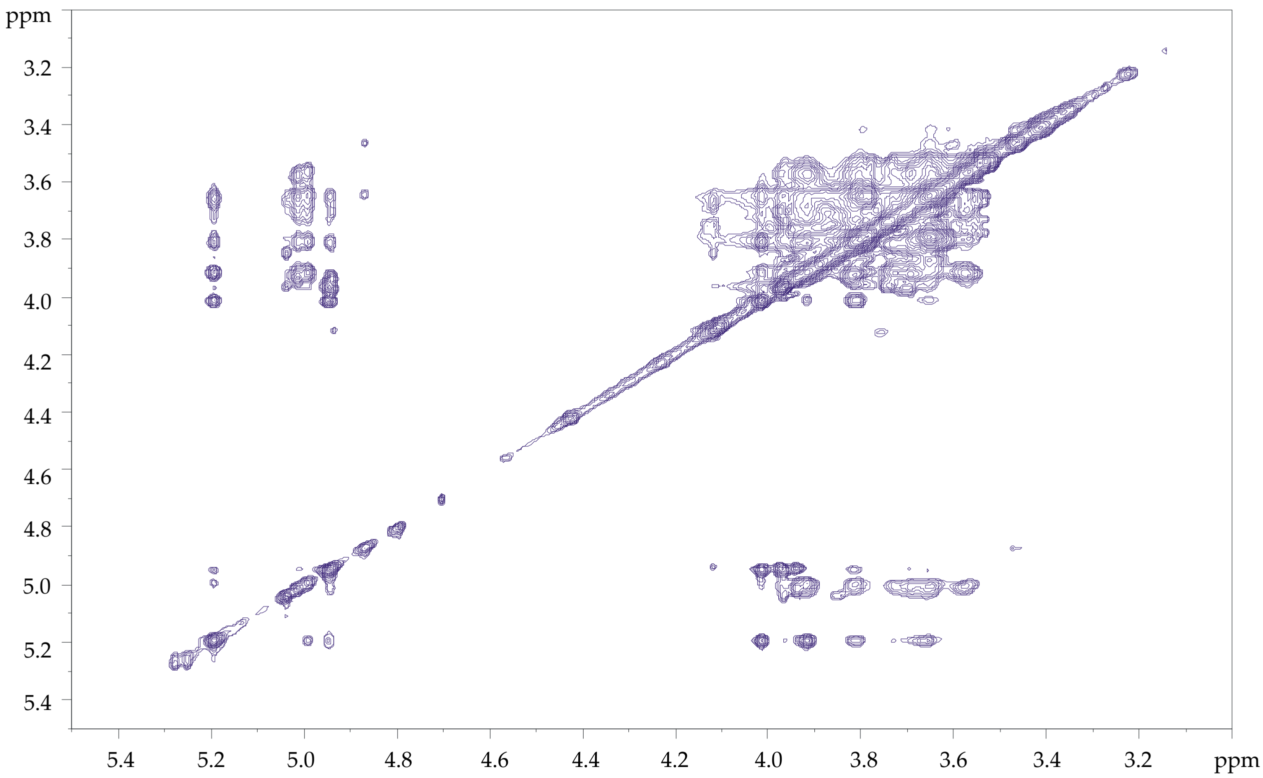

Figure S14. Expansion of the ^{1}H-^{1}H NOESY spectrum of Mannan$_{P.arc_HF}$ measured at 298 K in D_2O (150 ms as mixing time) at 600 MHz.

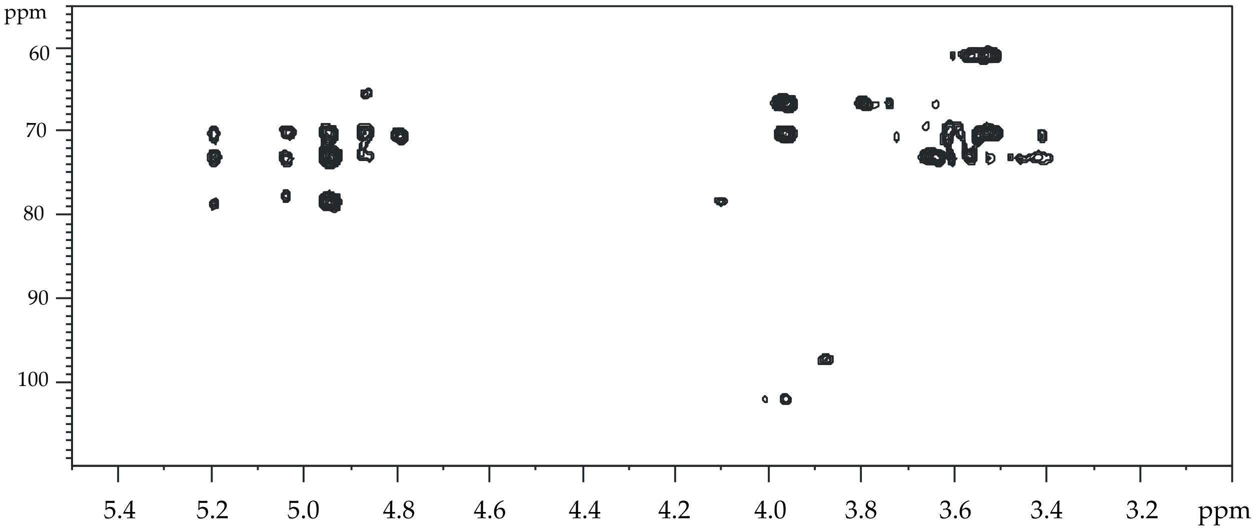

Figure S15. Expansion of the ^{1}H-^{13}C HMBC spectrum of Mannan$_{P.arc_HF}$. The spectrum was recorded in D_2O at 298 K at 600 MHz.

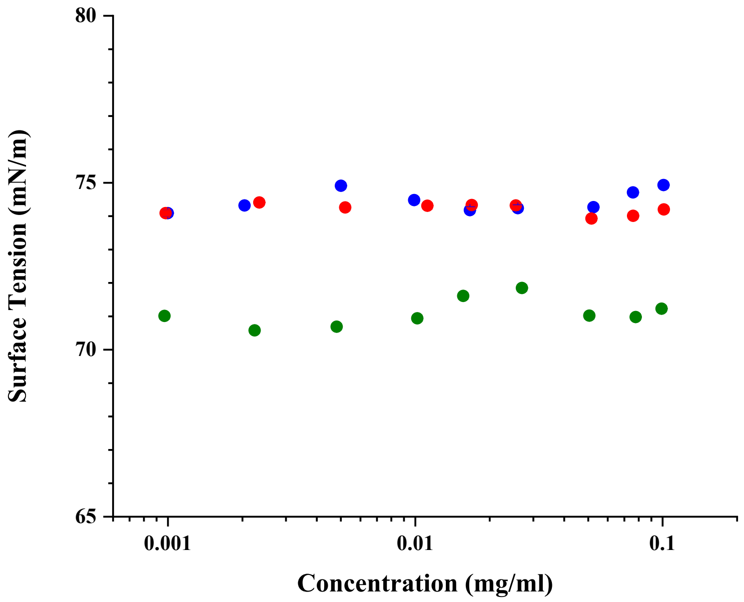

Figure S16. Surface tension as a function of Mannan$_{P.arc}$ (blue), Mannan$_{\text{Yeast,}}$ (green) and Mannan$_{P.arc_\text{HF}}$ (red) concentration at 25.0 °C.

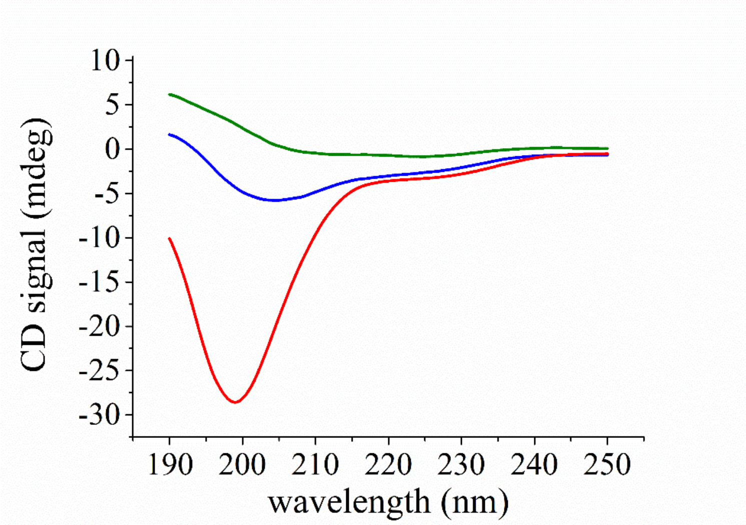

Figure S17. Overlapped CD spectra of Mannan$_{P.arc}$ (blue), Mannan$_{\text{Yeast}}$ (green) and Mannan$_{P.arc_\text{HF}}$ (red) at 20 °C.

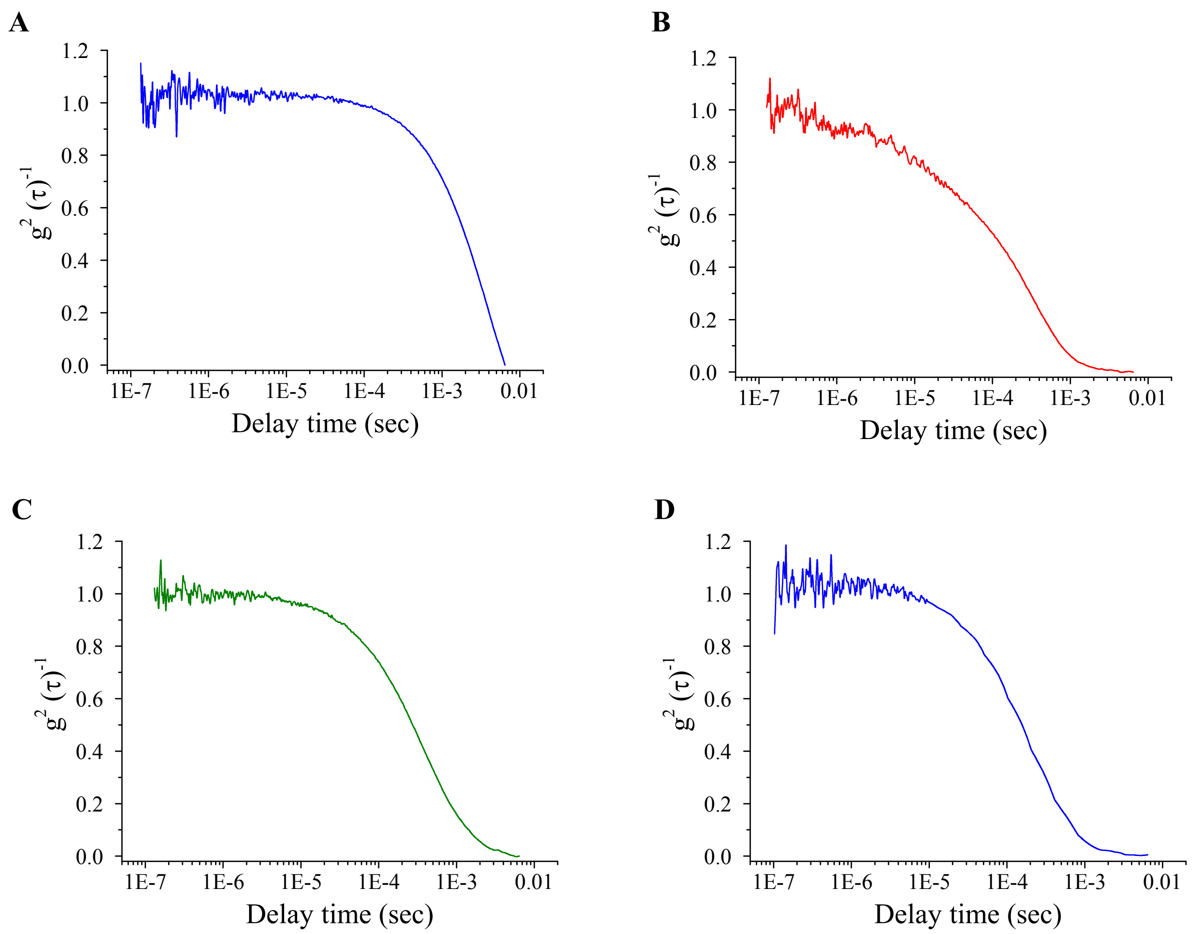

Figure S18. Normalized correlation functions for Mannan$_{P.arc}$ (A), Mannan$_{\mathrm{Yeast}}$ (B) and Mannan$_{P.arc_\mathrm{HF}}$ (C) at 6.3, 6.0 and 5.6 mg mL^{-1}, respectively, and for Mannan$_{P.arc}$ at 1 mg mL^{-1} (D).

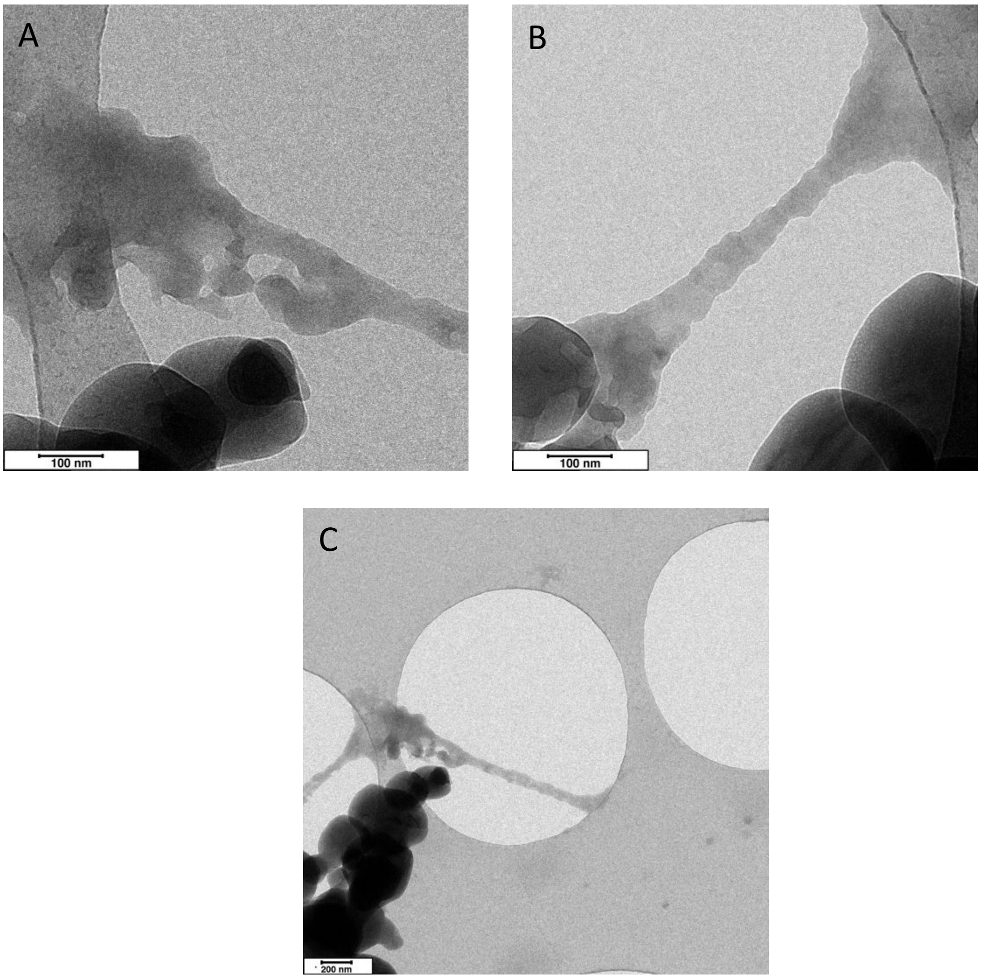

Figure S19. Cryo-TEM images collected on Mannan$_{P.arc}$.

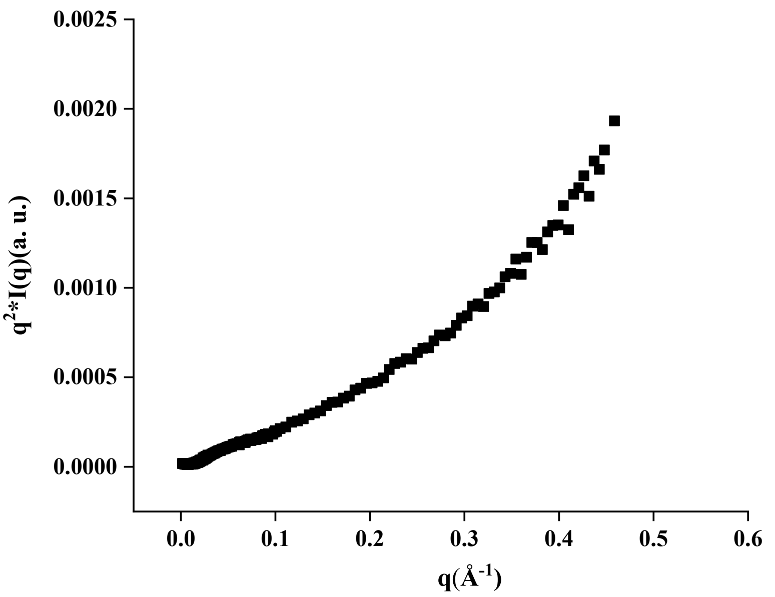

Figure S20. Kratky plot of SANS data for $Mannan_{Yeast}$.

Table S1. ^{1}H and ^{13}C NMR assignments of $Mannan_{P.arc\_HF}$. Spectra were recorded in D_2O at 298 K at 600 MHz using acetone as external standard (δ_H/δ_C 2.25/ 31.45 ppm).

**Sugar Residue**	$^{1}H/^{13}C$					
	1	2	3	4	5	6
**A**	5.19	4.01	3.81	3.63	3.66	3.64-3.79
2-Man*p*	101.8	79.7	71.6	68.1	74.5	62.3
**B**	5.04	3.96	3.75	3.53	3.66	3.64-3.78
t-Man*p*	103.5	71.3	71.6	68.2	74.5	62.4
**C**	5.01	3.93	3.83	3.73	3.70	3.58-3.89
2,6-Man*p*	99.4	80.0	71.4	67.8	72.1	66.9
**D**	4.99	3.91	3.81	3.73	3.70	3.58-3.89
2,6-Man*p*	99.4	80.0	71.5	67.8	72.1	66.9
**E**	4.94	3.96	3.70	3.53	3.66	3.64-3.79
t-Man*p*	103.4	71.3	71.7	68.1	74.4	62.4
**F**	4.93	4.11	3.84	3.72	3.69	3.64-3.79
3-Man*p*	103.5	70.9	79.2	67.6	72.5	62.4
**G**	4.79	3.88	3.73	3.59		3.64-3.87
6-Man*p*	100.6	71.3	71.7	68.1	72.1	66.7
**G'**	4.86	3.46	3.60	3.60	3.41	3.65-3.87
6-Man*p*	99.0	72.7	74.6	68.0	70.8	66.7
**I**	4.42	3.22	3.38	3.32	3.53	3.64-3.79
t-Glc*p*	104.3	74.4	76.7	70.9	76.1	62.4
